# Structural basis of cell wall peptidoglycan amidation by the GatD/MurT complex of *Staphylococcus aureus*

**DOI:** 10.1038/s41598-018-31098-x

**Published:** 2018-08-28

**Authors:** Erik R. Nöldeke, Lena M. Muckenfuss, Volker Niemann, Anna Müller, Elena Störk, Georg Zocher, Tanja Schneider, Thilo Stehle

**Affiliations:** 1 Interfaculty Institute of Biochemistry, University of Tübingen, D-72076 Tübingen, Germany; 2 Institute for Pharmaceutical Microbiology, University of Bonn, D-53115 Bonn, Germany; 3 Vanderbilt University School of Medicine, Nashville, Tennessee 37232 USA; 4 Present Address: Department of Biochemistry, University of Zurich, CH-8057 Zurich, Switzerland; 5 Present Address: Hain Lifescience GmbH, D-72147 Nehren, Germany

## Abstract

The peptidoglycan of *Staphylococcus aureus* is highly amidated. Amidation of α-D-isoglutamic acid in position 2 of the stem peptide plays a decisive role in the polymerization of cell wall building blocks. *S. aureus* mutants with a reduced degree of amidation are less viable and show increased susceptibility to methicillin, indicating that targeting the amidation reaction could be a useful strategy to combat this pathogen. The enzyme complex that catalyzes the formation of α-D-isoglutamine in the Lipid II stem peptide was identified recently and shown to consist of two subunits, the glutamine amidotransferase-like protein GatD and the Mur ligase homolog MurT. We have solved the crystal structure of the GatD/MurT complex at high resolution, revealing an open, boomerang-shaped conformation in which GatD is docked onto one end of MurT. Putative active site residues cluster at the interface between GatD and MurT and are contributed by both proteins, thus explaining the requirement for the assembled complex to carry out the reaction. Site-directed mutagenesis experiments confirm the validity of the observed interactions. Small-angle X-ray scattering data show that the complex has a similar conformation in solution, although some movement at domain interfaces can occur, allowing the two proteins to approach each other during catalysis. Several other Gram-positive pathogens, including *Streptococcus pneumoniae, Clostridium perfringens* and *Mycobacterium tuberculosis* have homologous enzyme complexes. Combined with established biochemical assays, the structure of the GatD/MurT complex provides a solid basis for inhibitor screening in *S. aureus* and other pathogens.

## Introduction

*Staphylococcus aureus* is a frequent constituent of human nasal microflora and a major cause of severe endogenous infections^1^. Effective treatment of staphylococcal infections remains a worldwide challenge. In the United States alone, Staphylococci are responsible for about 19,000 deaths per year, a number that is higher than that associated with HIV^2^. Methicillin-resistant Staphylococcus aureus (MRSA) strains, which are resistant to many commonly used antibiotics including methicillin, amoxicillin, penicillin, and oxacillin, represent an increasing challenge to human health worldwide^3^.

Species-specific cell wall modifications impact on several key aspects of the infection process, including adherence^1,4^, immune recognition^5^, and resistance to host defenses^6,7^. In Gram-positive bacteria such as *S. aureus*, a thick multilayered peptidoglycan (PG) layer constitutes the major component of the cell wall. The PG is essential for survival and maintenance of cell shape and is crucial to resist osmotic pressure^8^. The PG heteropolymer consists of alternating disaccharide units composed of N-acetyl-glucosamine (GlcNAc) and N-acetyl-muramic acid (MurNAc), which are cross-linked by short peptides to generate a rigid network.

Assembly of PG is a multistep process that begins in the cytoplasm and terminates on the exterior of the cell (Supplementary Fig. S1). The process is initiated by the MurA-F ligases, which catalyze the formation of the soluble PG precursor UDP-MurNAc-pentapeptide in the cytoplasm^9^. The membrane-bound enzyme MraY then links this precursor to the membrane carrier undecaprenyl phosphate to yield Lipid I (undecaprenyl-phosphate-MurNAc-pentapeptide), which is then connected with UDP-GlcNAc to form Lipid II (undecaprenyl-phosphate-MurNAc-pentapeptide-GlcNAc) by the glycosyltransferase MurG^10^. In *S. aureus*, Lipid II is modified by a Gly_5_-interpeptide bridge attached by the FemXAB peptidyltransferases^11,12^, followed by translocation across the cytoplasmatic membrane likely facilitated by the flippases FtsW, RodA and MurJ^13^. Once it has reached the exterior cell surface, the modified Lipid II is assembled into the growing PG network by penicillin-binding proteins (PBPs) catalyzing transglycosylation and transpeptidation reactions^14,15^. In particular, transpeptidation has been proposed to require at least one amidated stem peptide^16,17^.

Amidation of the α-carboxyl group of the D-isoglutamate residue in Lipid II, resulting in the formation of D-isoglutamine^16,18^, is catalyzed by a recently identified enzyme complex^19,20^. This complex consists of two proteins, GatD and MurT, which assemble into a binary complex. GatD has sequence similarities to the catalytic domains of glutamine amidotransferases, while MurT is similar in sequence to the substrate-binding domains of Mur ligases. Together, these two proteins catalyze the amidation of α-D-isoglutamic acid of cell wall precursor stem peptides in an ATP-dependent reaction. Mutants that are deficient in Lipid II amidation show a reduction in PG cross-linking and are more susceptible to antibiotics^21–23^. Thus, intervening with the amidation reaction in *S. aureus* may represent a useful strategy to combat this pathogen.

In order to provide insight into the overall organization of this complex and to facilitate an understanding of the amidation mechanism, we have determined the crystal structure of the GatD/MurT complex. We find that the two proteins assemble into a curved, boomerang-shaped structure, with GatD docking to the C-terminal domain of MurT. Together with mutagenesis data and structural analysis of a complex with an ATP analog, our data provide an excellent foundation to understand the concerted activities of both proteins. Small-angle X-ray scattering (SAXS) experiments confirm that the complex has a similar open conformation in solution, and suggest that some flexibility between the domains exist. Structure-based sequence alignments demonstrate that several other pathogenic organisms have homologous enzyme complexes that likely function in the same manner. In combination with the established *in vitro* assays, our findings provide the basis for more directed inhibitor screenings.

## Results

### Formation and characterization of the GatD/MurT complex

Full-length GatD and MurT were co-expressed as described^19^, and the complex was purified using nickel affinity chromatography. A final size exclusion chromatography step demonstrated that the two proteins elute together, forming a stable complex in solution. The elution volume in size exclusion chromatography corresponds to an estimated molecular weight of 72 kDa, which is consistent with the calculated molecular weight of 78.8 kDa for a binary GatD/MurT complex. The SAXS data (see below) also clearly indicate that one copy of GatD and one copy of MurT assemble into a stable heterodimer.

### Overall structure of GatD/MurT

**Figure 1:**
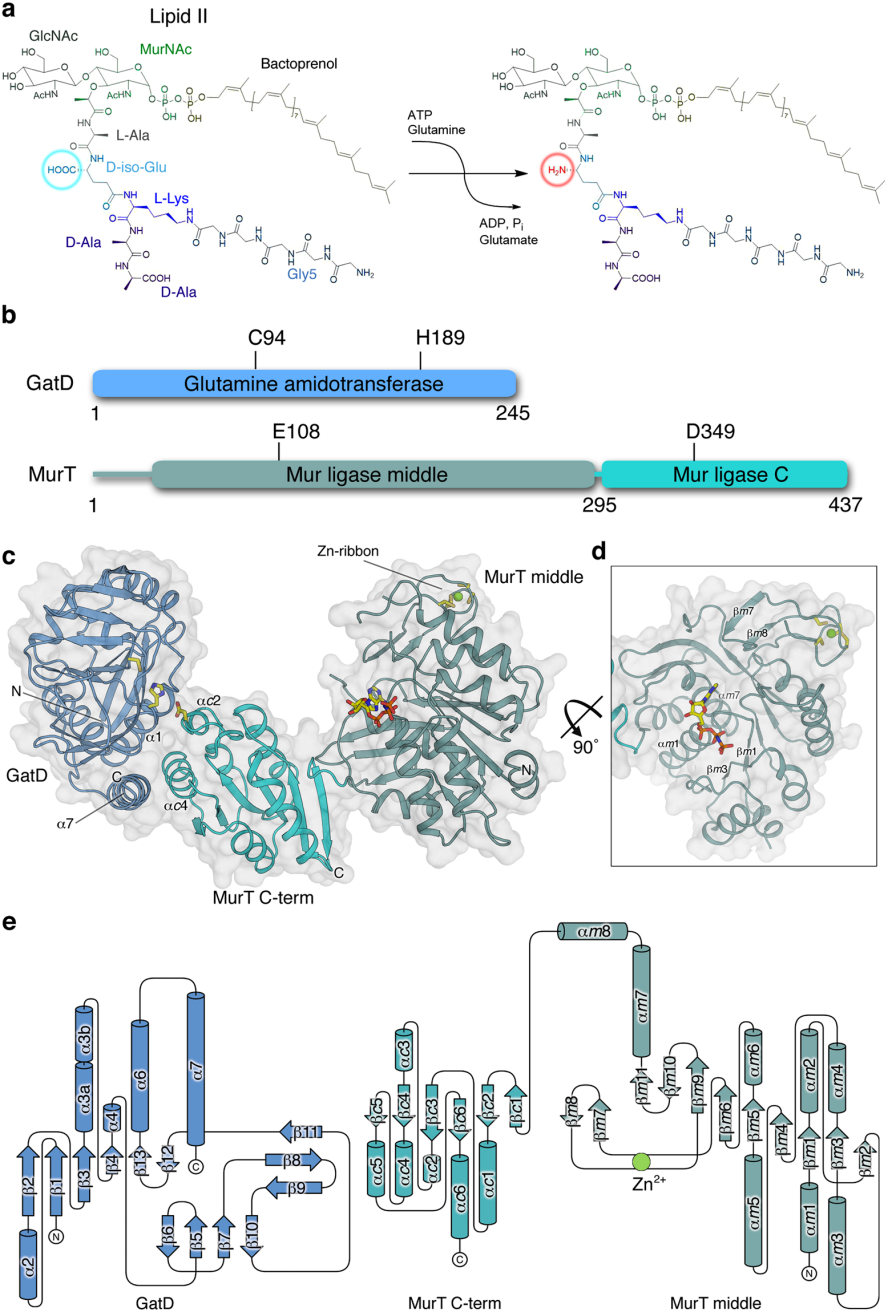
Overall structure and organization of the GatD/MurT complex. (**a**) Reaction catalyzed by GatD/MurT. The free α-carboxyl of D-iso-glutamate in the peptide stem is amidated in a glutamine- and ATP-dependent reaction. (**b**) Schematic overview of GatD and MurT proteins. GatD consists of a single glutamine amidotransferase (GATase) domain with a cysteine at position 94 as the active residue and a histidine at position 189 as a component of the catalytic triad^19^. MurT is composed of two domains: a Mur ligase middle domain (MurT middle) containing the canonical ATP binding site and, surprisingly, a ribbon-type Zinc finger, and a C-terminal Mur ligase domain (MurT C-term). MurT residue glutamate 108 participates in ATP hydrolysis, and aspartate 349 forms the third residue in the putative catalytic triad. (**c**) Overview of the GatD/MurT structure. GatD and MurT form a boomerang-shaped complex, with GatD contacting the MurT C-term domain through contacts that are in part mediated by helix α7 of GatD. Catalytic triad residues GatD-C94, GatD-H189, MurT-D349 and the bound nucleotide AMPPNP are shown in stick representation. The zinc ion in the Cys_4_ zinc ribbon of MurT is shown as a green sphere, and the four cysteine residues ligating it are shown as sticks. (**d**) Tilted view of the MurT middle domain to show the central β-sheet and the bound AMPPNP and its surrounding secondary structure elements, as well as the zinc ribbon. (**e**) Topological representation of the GatD/MurT architecture. Secondary structure nomenclature of GatD was done according to Leisico *et al*.^24^. As the short helices α1 and α5 in the isolated GatD structure do not conform to helical geometry in our complex, they were not assigned. The MurT domains were assigned separately with the prefixes *m* and *c* indicating the middle and C-terminal domains, respectively. The drawing was generated with TopDraw^54^.

The native structure of the GatD/MurT complex was solved at a resolution of 2.04 Å using single isomorphous replacement with anomalous scattering (SIRAS). The refined structure has excellent statistics (Table 1) and includes all residues of the expressed proteins with the exception of MurT residues 1–35, 195–196 and 434–437. These regions are poorly visible in the electron density maps and therefore likely have multiple conformations and increased mobility. The GatD/MurT heterodimer adopts a boomerang-shaped conformation, with GatD packing against the C-terminal domain of MurT (Fig. 1). As previously postulated^19^ and recently shown^24^, GatD exhibits a class-I glutamine amidotransferase-like fold. A DALI search^25^ identifies the enzymes HisH, PdxT and PurQ from *Thermotoga maritima* as the closest structural homologs (Z-values of 17.4, 15.8 and 15.0, respectively). Superimposition of GatD with structures obtained from a secondary structure-based search using HHPRED^26^ reveals a well-conserved core architecture, with root-mean-square deviation (r.m.s.d.) values of 2.6 Å (all C-α atom pairs) over the entire length of GatD for the closest structural homolog, HisH. However, GatD distinguishes itself from other, homologous structures through the presence of an extended C-terminal helix, termed helix α7 (Fig. 1c). This helix mediates many of the contacts with MurT, explaining its presence in GatD.

**Table 1:** Crystallographic data and refinement statistics.

Dataset (PDB ID)	native (6GS2)	thiomersal	AMPPNP (6H5E)
**Data collection**			
X-ray source	SLS X06DA (PXIII)	SLS X10SA (PXII)	SLS X06DA (PXIII)
X-ray detector	Pilatus 2 M	Pilatus 6 M	Pilatus 2 M
Wavelength [Å]	1.0	1.0	1.0
Space group	P2_1_2_1_2_1_	P2_1_2_1_2_1_	P2_1_2_1_2_1_
Unit cell axes (Å)	a = 107.10 b = 110.37 c = 116.36	a = 106.97 b = 109.27 c = 116.03	a = 109.72 b = 109.74 c = 123.30
Unic cell angles (°)	α = β = γ = 90	α = β = γ = 90	α = β = γ = 90
Resolution [Å]	50–2.04	50–2.49	50–2.14
Reflections (unique)	641308 (88009)	1411718 (91196)	1114473 (82581)
Redundancy	7.3	13.8	13.5
Complenteness [%] (last bin)	99.7 (99.1)	99.7 (98.1)	99.8 (98.6)
I/σ(I)	14.01 (1.57)	17.33 (1.88)	19.37 (1.41)
R_meas_ [%]	11.2 (178.9)	3.5 (131.3)	11.6 (213.0)
CC_1/2_ [%]	99.9 (72.5)	99.9 (71.2)	100.0 (67.6)
Wilson B [Å^2^]	40.9	56.0	51.9
**SIRAS phasing**			
Resolution [Å]		50–2.08	
No. of heavy atoms in ASU		13	
Phasing power (iso/ano)			
centric		0.898/−	
acentric		0.685/0.382	
FOM			
centric		0.15862	
acentric		0.14178	
Rcullis (iso/ano)			
centric		0.856/−	
acentric		0.886/0.965	
**Refinement**			
Resolution included [Å]	49.07–2.04		49.08–2.14
Software	PHENIX (1.10.1)		PHENIX (1.10.1)
Non-solvent atoms	9893		9909
Solvent atoms	639		442
R_work_/R_free_ [%]	17.5**/**21.6		19.2**/**23.5
Size of R_free_ test set [%]	1.71 (1503 reflections)		1.82 (1504 reflections)
Bond r.m.s.d.	0.011		0.011
Angle r.m.s.d.	1.097		1.424
Ramachandran [%] (favoured, allowed, outliers*)	97.43, 2.57, 0.00		96.44, 3.48, 0.08
Rotamer outliers [%]	1.97		1.79
All-atom clashscore^+^	3.34		5.02
Average B factors [Å^2^]	41.3		60.2
protein	41.0		60.1
ligand	—		50.4
ions	42.8		59.3
solvent	45.6		63.8

^*^Outliers are residues C94 and G190 in each of the two copies of GatD. Both have well-defined density.

^+^PDB validation reports show that these values are comparable to or better than those of structures with similar resolution.

MurT contains the Mur ligase middle and C-terminal domains typical for the Mur ligase family. The C-terminal domain is built around a central six-stranded, predominantly parallel β-sheet (Fig. 1c,e) that is sandwiched between four α-helices on one (α*c*1–4) and two α-helices (α*c*5-6) on the other side. The middle domain is constructed around a crescent-shaped, nine-stranded predominantly parallel β-sheet, which encloses a three-helix bundle (α*m*1, α*m*7-8). The ATP molecule required for catalysis, replaced here by the non-hydrolyzable analog β,γ-imidoadenosine 5′-triphosphate (AMPPNP), is bound at the base of helix α*m*1 with the adenine moiety bound in a cleft formed next to helix α*m*1 while the phosphates are contacted by residues from strands β*m*1 and β*m*3 (Fig. 1d). The central β-sheet is capped on the opposite side by five α-helices. A RanBP-type Zinc-ribbon^27^ is located on the side of the middle domain as an insertion between β-strands β*m*6 and β*m*9. This feature is missing in homologous proteins. A search with DALI^25^ yielded MurF enzymes from *Pseudomonas aeruginosa*, *Escherichia coli* and *Acinetobacter baumannii* as the closest structural homologs, with Z-values of 23.2, 22.6, and 21.7, respectively. Mur ligases typically contain a third, N-terminal domain, which is not present in MurT. Instead, MurT only contains a truncated and likely flexible N-terminus (residues 1–37), which is not visible in our electron density maps.

### Overall conformation

It can sometimes be challenging to assign correct physiologic contacts from crystal structures alone as crystal packing can offer alternative solutions for possible contacts between two subunits that are often not easy to distinguish from physiologic contacts^28^. In order to validate the observed interaction between GatD and MurT, all possible GatD/MurT interfaces in the crystal were analysed with the PISA^28^ and EPPIC^29^ servers. Both algorithms clearly classify only the interaction depicted in Fig. 1c as physiologic. Other possible interactions between GatD and MurT have much smaller buried surface areas and are classified as crystal packing artifacts. Moreover, they do not place critical catalytic residues (see below) in close proximity.

**Figure 2:**
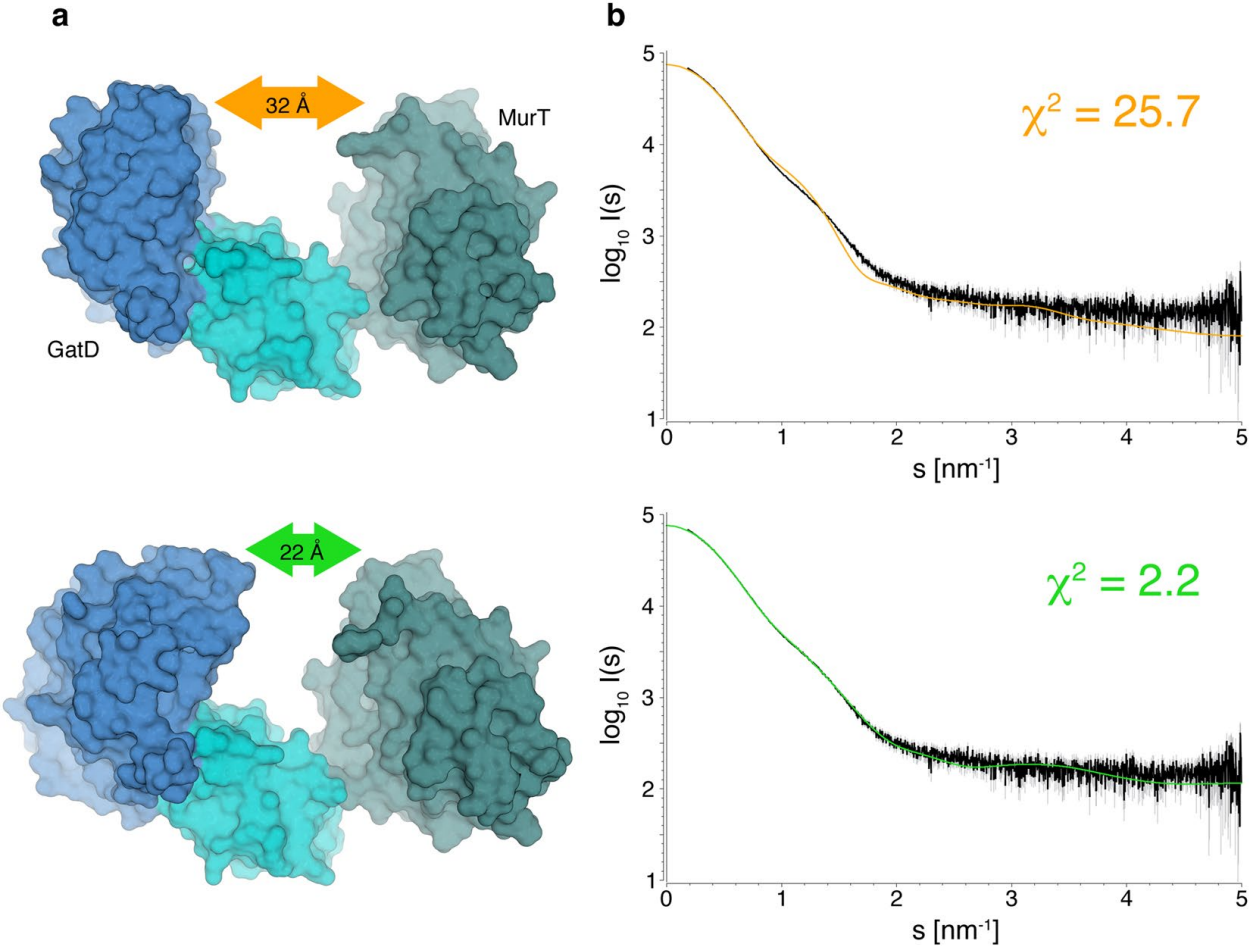
Conformation of the GatD/MurT complex in solution. (**a**) GatD/MurT crystal structure before (top) and after (bottom) refinement against SAXS data. While the overall organization and shape of the complex remain similar, the increased diameter of the GatD and MurT middle domain envelopes as well as the smaller gap of 22 Å instead of 32 Å between them suggest that some flexibility between domains exists in solution. (**b**) Fit of synthetic scattering profiles derived from the crystal structure (top) and refined model (bottom) against the experimental SAXS data.

The overall structure of the GatD/MurT complex is also supported by solution SAXS experiments (Fig. 2). Scattering profiles derived from dilution series experiments were used to refine the crystallographic model. After rigid body refinement, the overall fit of a calculated scattering curve to the experimental data improved from an initial χ^2^ = 25.7 to χ^2^ = 2.2 (Fig. 2b). The refined model retains the overall characteristics of the crystal structure, but it has a more compact conformation with a closest distance between GatD and the MurT middle domain, measured at the top of the structure, of 22 Å instead of 32 Å (Fig. 2a). These experiments show that, while the conformation of the unliganded GatD/MurT complex is indeed open and elongated as observed in the crystal structure, the complex can breathe somewhat in solution. Unfortunately, SAXS data obtained from titrations with AMPPNP and a soluble Lipid II mimic (UDP-MurNAc-L-Ala-D-Glu-γ-L-Lys-D-Ala-D-Ala) resulted in moderate to severe aggregation of GatD/MurT (Supplementary Fig. S2) and could thus not be used for modelling. However, the observed change in protein solubility clearly suggests a considerable conformational rearrangement in the protein upon ATP and Lipid II binding.

### Interactions between GatD and MurT

**Figure 3:**
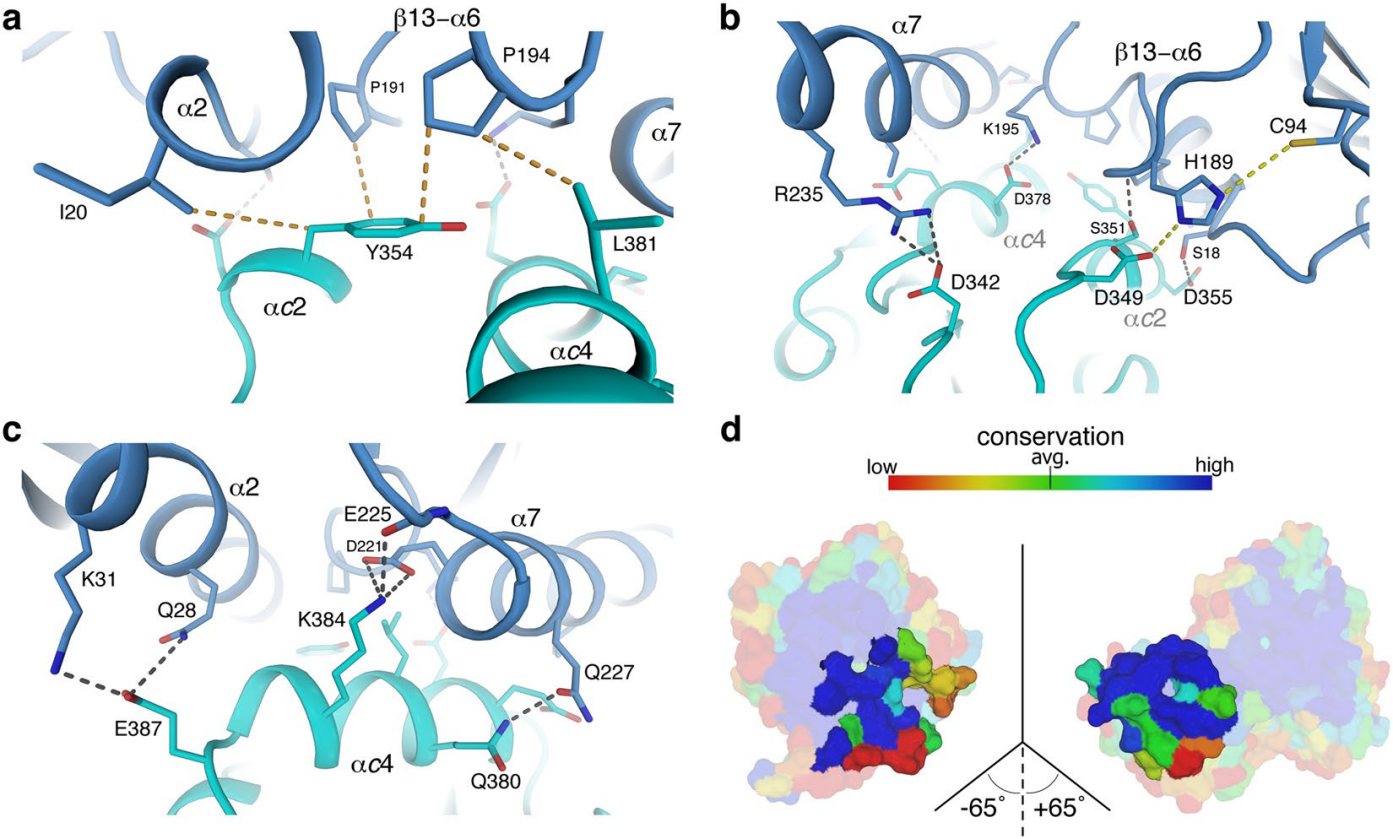
Architecture of the interface between GatD and MurT. (**a**) Hydrophobic interactions at the core of the GatD-MurT interface, centered around MurT-Y354. (**b**) and (**c**) Views of the rim of the interface showing mainly polar contacts or salt bridges. The C-terminal helix of GatD, which is unique to these types of proteins, is labeled helix α7, and the core hydrophobic loop was termed β13-α6. (**d**) Open-book view of the interface between GatD (left) and MurT (right). Sequence conservation scores were calculated with ConSurf^34^ and mapped onto the protein surface as described in the methods section. The coloring scheme is also shown at the top of the panel. The majority of the interactions between GatD and MurT are very highly conserved, as indicated by dark and light blue colors.

Extensive interactions between GatD and MurT establish a large, continuous interface that buries a total surface area of 940 Å^2^ from solvent (calculated with PISA^28^). Residues involved in interface formation are contributed by two consecutive MurT α-helices (α*c*2/3 and α*c*4), which tightly pack against two α-helices and a loop of GatD (helices α2 and α7, loop β13-α6 Figs 1 and 3). One of these α-helices is the C-terminal helix α7 absent in homologous class-I glutamine amidotransferases, which do not form similar complexes with MurT. The interface is entirely devoid of solvent molecules. Two large aromatic (GatD-F146, MurT-Y354) and several aliphatic side chains (I20, I24, P194, and V231 in GatD and I353, L381 and L385 in MurT) that would otherwise be solvent-exposed are buried upon formation of the GatD/MurT contact. Figure 3a shows interactions made by the MurT-Y354 and MurT-L381 side chains as an example. MurT-Y354 also contacts two prolines in the GatD β13-α6 loop (P191 and P194), with P191 being especially well conserved throughout GatD homologs but not across functionally unrelated glutamine amidotransferases. These more centrally located hydrophobic interactions are augmented by intermolecular salt bridges (GatD-K31/MurT-E387, GatD-E225/MurT-K384, GatD-K195/MurT-E378, GatD-R235/MurT-D342), which are located towards the edges of the interface, and by hydrogen bonds (Fig. 3b,c). Residues participating in interface formation are highly conserved across species that have homologous proteins, as demonstrated by the conservation analysis shown in Fig. 3d. The interface also contains several residues with particularly small side chains (G21, A25, G190, A224, and A228 in GatD and S351 in MurT), and in each case a larger side chain would be incompatible with the observed interface.

### Catalytic site of GatD

**Figure 4:**
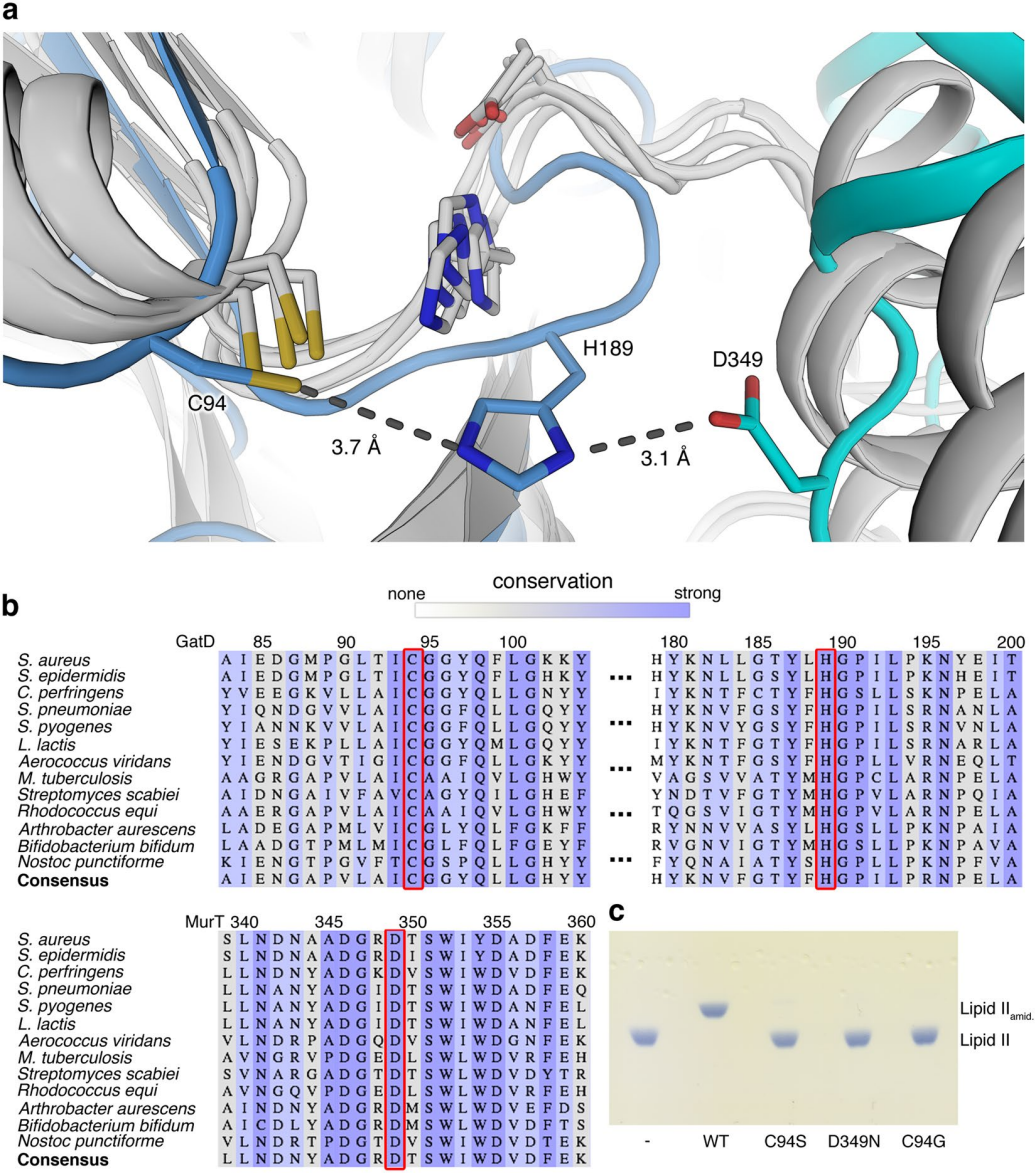
The putative catalytic triad of GatD/MurT. (**a**) Overlay of GatD/MurT with four glutamine amidotransferase fold-containing proteins obtained from a HHPRED^26^ search (white cartoon and sticks). Whereas most GATases possess a conserved catalytic triad consisting of cysteine, histidine and glutamate residues, the glutamate is replaced by a proline (GatD-P191) in the GatD sequence and the conserved histidine (GatD-H189) is oriented towards an aspartic acid (MurT-D349) in MurT. (**b**) Multiple sequence alignment of putative homologous GatD/MurT enzymes. Conservation is color-coded, with white indicating low conservation, grey medium, and dark blue indicating high conservation. Residues GatD-C94, GatD-H189 and MurT-D349 are highly conserved (red box), as well as their immediate surroundings. (**c**) Thin-layer chromatography analysis of an activity assay of catalytic triad mutants. Mutation of MurT-D349 to asparagine completely abolishes catalysis *in vitro*, similarly to GatD-C94 mutations to either serine or glycine.

Class-I glutamine amidotransferases contain a highly conserved catalytic triad in their active site, which consists of a cysteine responsible for the initial nucleophilic attack on the substrate and nearby histidine and glutamic acid residues to form a proton relay chain. Even remotely related members of this enzyme family superimpose very well for the active site (Fig. 4). Earlier studies suggested that GatD residues C94 and H189 might form part of a catalytic triad^19^. In homologous GATases, the third residue of this triad is typically a glutamic acid that is located two residues downstream of the histidine in a conserved HPE tripeptide sequence^30^. This glutamic acid is replaced with a proline (P191) in GatD and separated from H189 by a glycine, resulting in a HGP motif that is well conserved throughout putative GatD enzymes. However, as proline cannot function as a proton relay in a catalytic triad, GatD enzymes must either function with only a catalytic dyad, as recently suggested^24^, or have arrived at a different solution to establish such a triad.

The structurally homologous enzymes PdxT from *Bacillus subtilis* (PDB ID 2nv0), two homoserine O-acetyltransferases from *Bacillus cereus* and *Thermotoga maritima* (PDB IDs 2vfj and 2h2w, respectively), and the glutamine amidotransferase Mfla_0438 from *Methylobacillus flagellatus* (PDB ID 3m3p), obtained from a HHPRED search, align well with the overall structure of GatD. A closer inspection of the active site architecture reveals that GatD-C94 superimposes well with the active site cysteine residues from these enzymes (Fig. 4a). The sulfhydryl groups in particular are located in almost identical positions. However, the side chain of GatD-H189 does not overlay well with the histidines from the related enzymes (Fig. 4a). Instead, GatD-H189 is shifted towards the heterodimer interface, where it contacts an aspartic acid (D349) of MurT, thus establishing a possible alternative catalytic triad (GatD-C94, GatD-H189, MurT-D349) involving residues from both GatD and MurT. A superimposition of our structure with the recently published structure of monomeric GatD^24^ (PDB ID 5n9m) shows that the interaction with MurT is required to fully position the loop containing GatD-H189 (Supplementary Fig. S3). The close proximity of the side chains of GatD-C94 and GatD-H189 (3.7 Å) and GatD-His189 and MurT-D349 (3.1 Å) as well as their relative orientation suggest that these three residues might indeed function as a proton relay system in catalysis. GatD-H189 is followed by a short hydrophobic sequence framed by two proline residues, GatD-P191 and GatD-P194, that are involved in contacts with MurT (Fig. 3a). The arrangement of the putative catalytic triad is therefore dependent on correct assembly of the binary complex. As shown in Fig. 4, sequence comparison with confirmed^31^ and predicted homologs^19^ as well as a sequence- and structure based conservation analysis revealed the combination of GatD-H189 and the subsequent hydrophobic stretch to be highly conserved, suggesting that the GatD homologs possess similar catalytic triads and interfaces with corresponding MurT homologs. Similarly, the sequence context of MurT-D349 is conserved within MurT homologs, but not across other Mur ligases.

In order to investigate whether the spatial proximity of MurT-D349 to GatD-C94 and GatD-H189 is indicative of a possible involvement in catalysis, a MurT-D349N mutant was generated. *In vitro* amidation assays of Lipid II revealed severely reduced *in vitro* activity of this mutant compared to the wildtype GatD/MurT complex (Fig. 4, Supplementary Fig. S4). A conservative mutation of the active site cysteine (GatD-C94S) to serine completely abolished activity *in vitro*, similarly to the previously characterized, less conservative GatD-C94G mutant^19^. The folds and thermal stabilities of the mutants were assessed with circular dichroism (CD) spectroscopy and thermal shift assay (TSA), respectively. Both MurT-D349N and GatD-C94S possess a fold indistingushable from that of the wildtype protein (Supplementary Fig. S5) as well as virtually identical melting temperatures (Supplementary Fig. S6, Table S1) that lie well above the temperature used for activity assays. Taken together, the mutagenesis experiments demonstrate that both GatD-C94 and MurT-D349 are likely relevant for catalytic activity of the complex, and they therefore also provide additional support for the physiologic nature of the observed GatD/MurT interface.

### The nucleotide binding site of MurT

**Figure 5:**
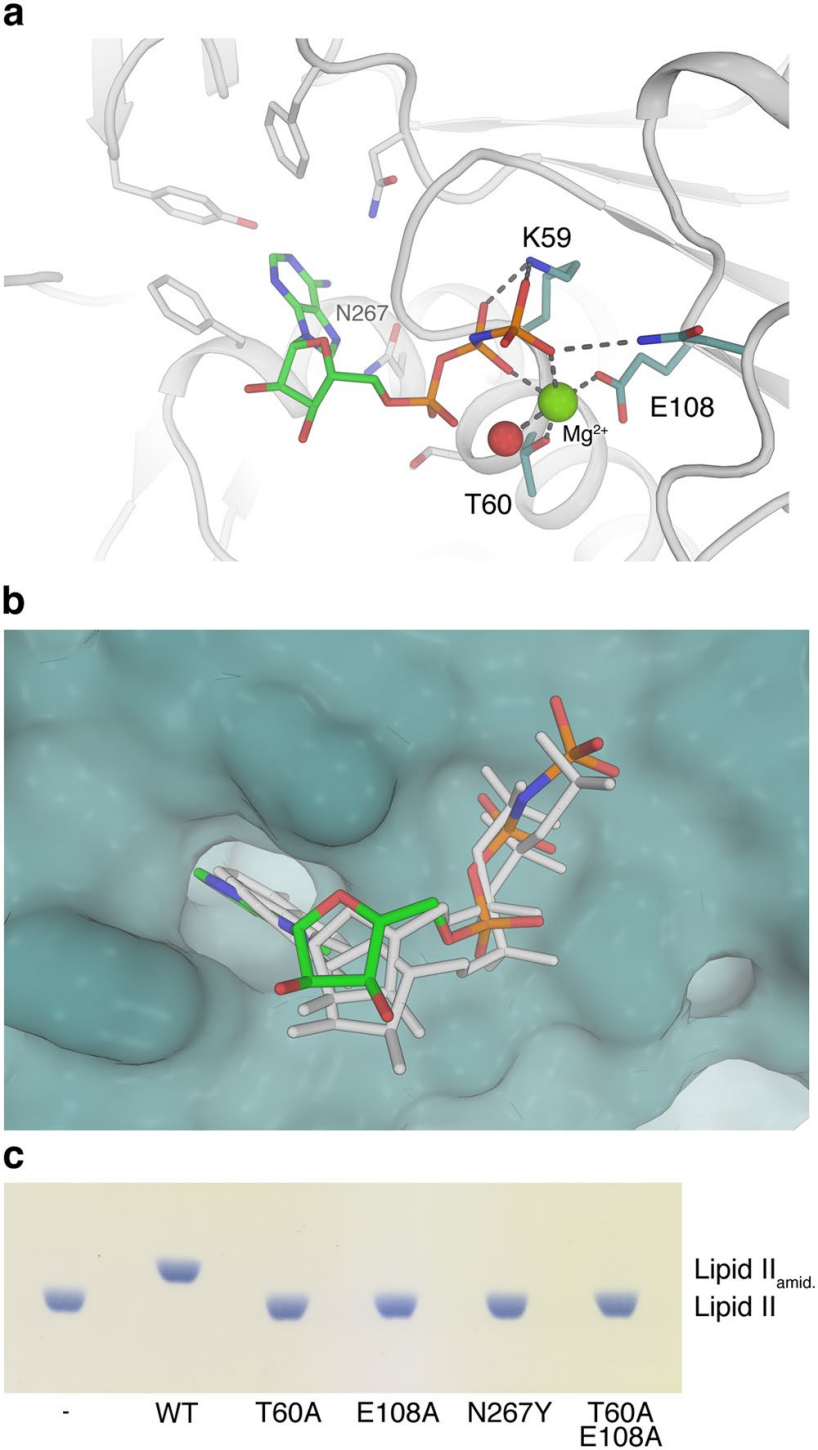
The AMPPNP binding site in GatD/MurT. (**a**) Catalytic center of MurT bound to the ATP analogue AMPPNP. The adenine base is inserted into a pocket composed of several aromatic residues and two asparagines, including N267, while the conserved K59, T60 and E108 residues coordinate the β and γ phosphates as well as a magnesium ion (green sphere) found in the active center of ATPases. A bound water is shown with a red sphere. (**b**) Superimposition of ATP analogues from *S. aureus* MurE (Protein Data Bank ID 4c12)^32^ and *P. aeruginosa* MurF (Protein Data Bank ID 4cvk) onto the MurT ATP-binding pocket in surface representation based on structural superimpositions of the entire domains. The MurT-bound AMPPNP is shown as a colored stick model, the superimposed nucleotides from the two related structures are shown as white sticks. (**c**) Thin-layer chromatography analysis of an activity assay of ATP-binding site mutants. Mutation of the magnesium-coordinating residues T60 and E108 to alanines completely abolishes catalysis. Replacement of the conserved N267 with a bulky tyrosine residue also impedes catalysis, probably by interfering with AMPPNP binding.

To obtain insight into interactions of the GatD/MurT complex with ATP, we prepared a ternary complex by soaking crystals with 2.1 mM of AMPPNP, an ATP analog. The structure of this complex was solved at 2.14 Å resolution using molecular replacement (Table 1), and the corresponding electron density allowed us to unambiguously model AMPPNP into a binding site in the middle domain of MurT (Figs 1d and 5, Supplementary Fig. S7). The binding site contains the consensus sequence GTNGKT^19^, with residues K59 stabilizing the β and γ phosphate groups of AMPPNP and T60 coordinating a magnesium ion that is also contacted by the side chain of the conserved MurT-E108 residue. The magnesium ion helps in positioning the AMPPNP β and γ phosphates from the opposite side (Fig. 5a). The position of the observed ATP binding pocket is strictly conserved throughout Mur ligases as structural superposition of the middle domain of MurT with the middle domains of close sequence homologues MurE from *S. aureus*^32^ (PDB ID 4c12) and MurF from *Acinetobacter baumannii*^33^ (PDB ID 4qdi) revealed nearly identical modes of nucleotide-binding (Fig. 5b). The observed binding mode is in excellent agreement with our mutagenesis data, as single or double mutants of nucleotide-binding site residues (T60A, E108A and N267Y) have completely lost their enzymatic activity (Fig. 5c, Supplementary Fig. S8). As with the previously described mutants, protein fold and stability were confirmed via CD spectroscopy and TSA (Supplementary Figs S5 and S6, Table S1).

### Conservation mapping

**Figure 6:**
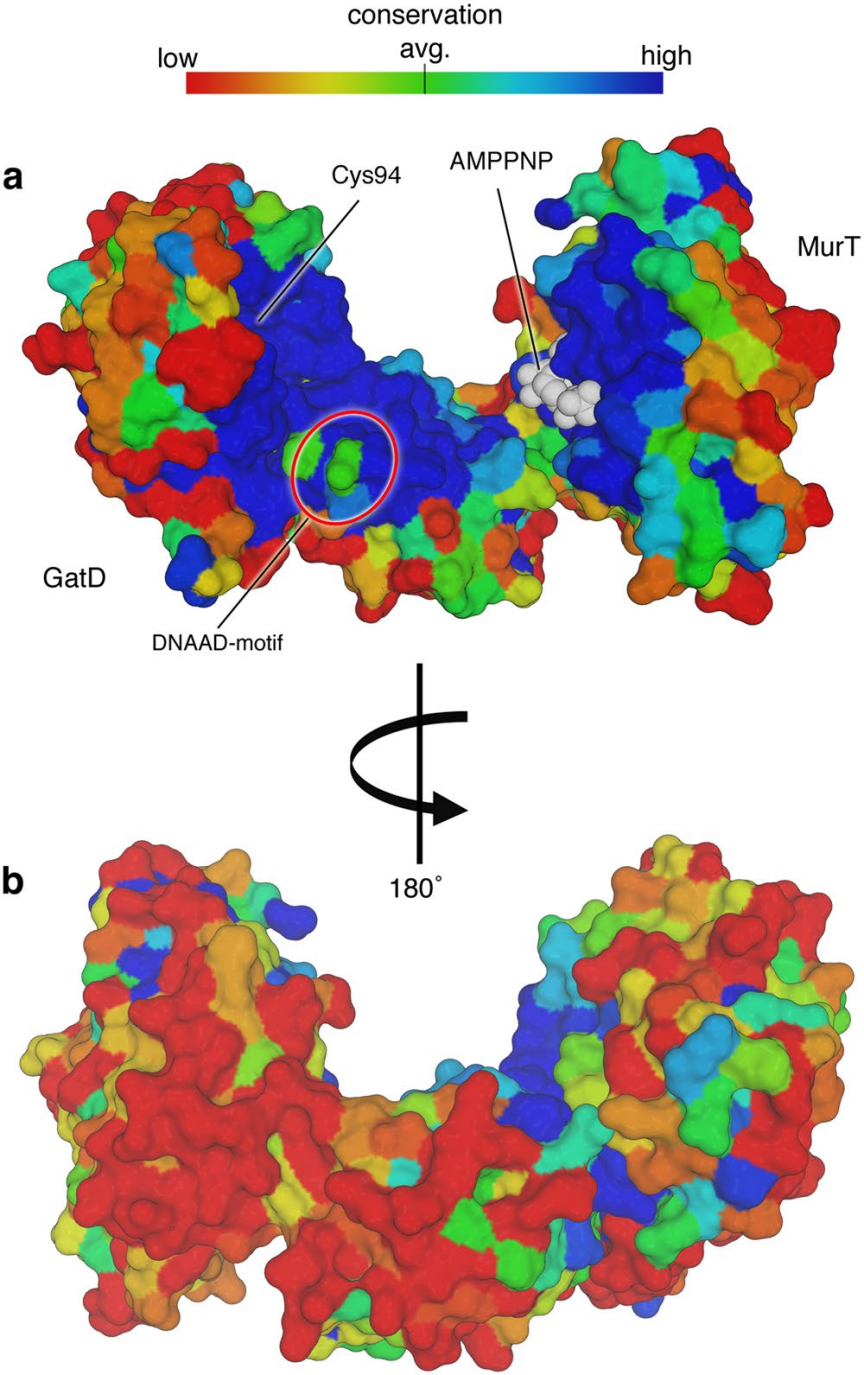
Conservation analysis of the GatD/MurT surface. (**a**) Inner and (**b**) outer surface of the GatD/MurT crystal structure colored by a color gradient indicating conservation, as shown on the top of the figure. Conservation scores were calculated with ConSurf^34^ and mapped onto the protein surface as described in the methods section. Dark blue signifies a high conservation score. Most of the concave surface of GatD/MurT is highly conserved, whereas the remaining surface displays high variability. The AMPPNP ligand is shown with white spheres, and the location of the catalytic triad and the conserved DNAAD sequence that has been implicated in muropeptide binding are indicated as well.

Conservation analysis based on a redundance-corrected alignment of 145 homologous sequences, as automatically performed on the ConSurf server^34^ and projected onto the surface of the GatD/MurT model, revealed a high level of conservation at the inside of the crescent-shaped binary complex. While the outwards-facing surface of the protein, including that of the Zinc finger, is variable in sequence, the inner surface residues, which contain the ligand-binding regions of other Mur ligases, are strongly conserved. This includes the aforementioned components of the putative catalytic triad composed of GatD-C94, GatD-H189 and MurT-D349, as well as the MurT-GTNGKT nucleotide binding motif and MurT-E108 (Fig. 6), which coordinates the magnesium ion that lies in proximity to the bound ATP analog. The consensus sequence D(D,N)P(N,A) in the amino acid‐binding pocket of MurE from *Thermotoga maritima* was found to mediate the recognition of L-lysine^35^. Structural work by Ruane and colleagues revealed that the *Staphylococcus aureus* MurE protein contains additional, often charged residues that are important for the interaction with L-lysine, e. g. Asp-406 and Glu-460^32^.

**Figure 7:**
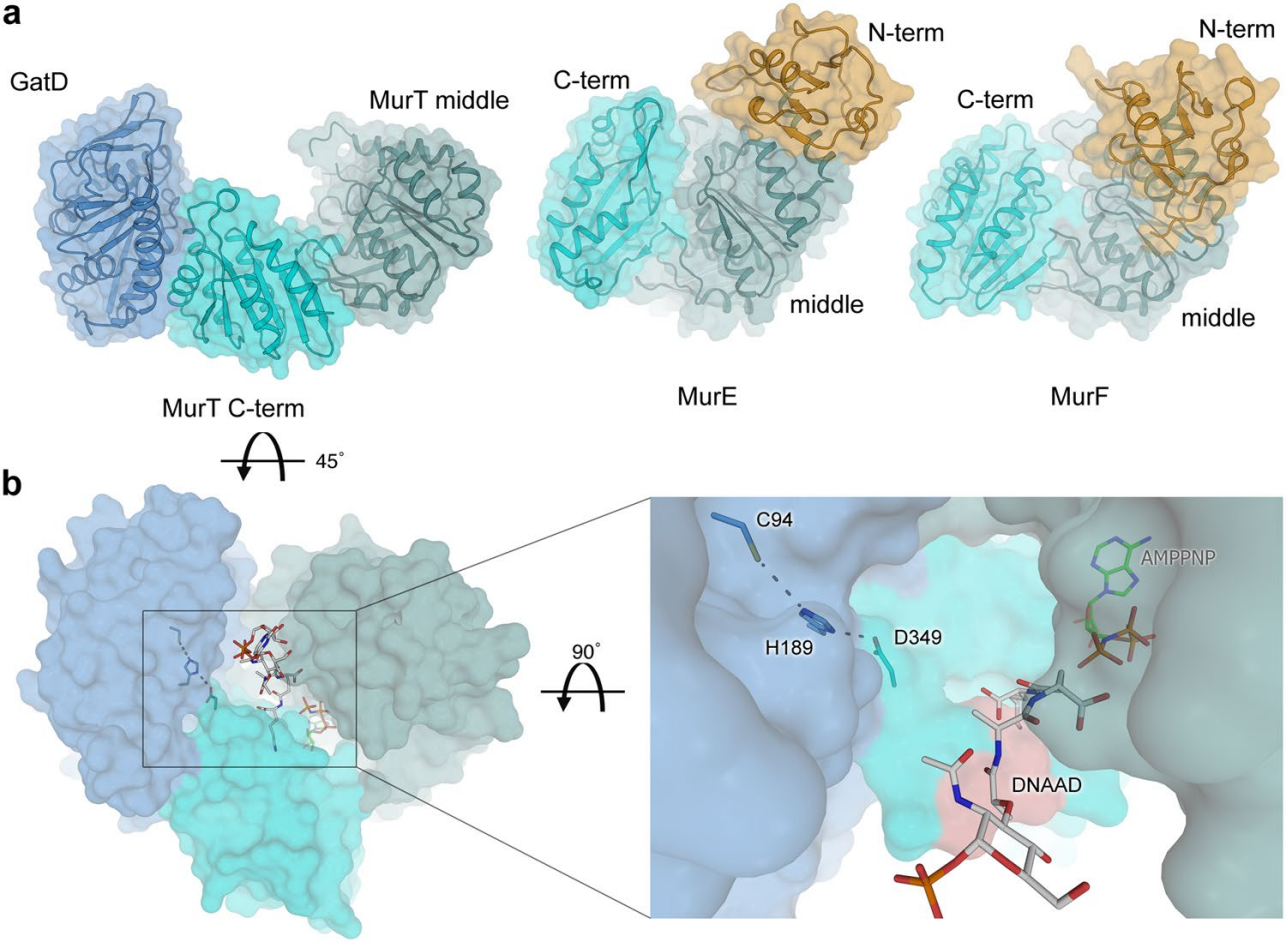
Comparison of GatD/MurT with Mur ligases and putative conformational changes upon ligand engagement. (**a**) Structural alignment of GatD/MurT (left) and *S. aureus* MurE^32^ and *A. baumannii* MurF^33^ structures (right) was performed based on the Mur ligase middle domain (light green). The C-terminal domain (cyan) is rotated toward the middle domain in the MurE and MurF structures. A UDP-binding N-terminal domain (orange) is present in MurE and MurF but not in MurT. (**b**) Putative model of an active conformation of GatD/MurT with the domain movement modeled after the *S. aureus* MurE structure. The GatD catalytic triad and the bound AMPPNP molecule are shown as colored sticks, the relevant muramyltripeptide portion of the superimposed substrate of MurE (MurNAc-L-Ala-γ-D-Glu-L-Lys, but lacking UDP) is shown in grey stick representation for reference. The DNAAD motif, suggested to be involved in substrate binding is highlighted in salmon (right).

The MurT sequence DNAAD containing the conserved aspartate residue was previously suggested to be involved in interactions with the lysine residue of the Lipid II stem peptide^19^. Conservation mapping revealed that this motif is conserved and located in a large, concave area between the GatD catalytic triad and the ATP binding site in the middle domain of MurT (Fig. 7). Thus, it seems reasonable that this region is functionally relevant, either by engaging parts of Lipid II or by mediating conformational changes.

## Discussion

The recently characterized GatD/MurT complex is responsible for the amidation of glutamic acid in the peptide stem structure of the *S. aureus* PG^19^. Inhibition of this reaction leads to lower growth rate, reduced resistance to beta-lactam antibiotics, and increased sensitivity to lysozyme in MRSA strains^19,20^. Strategies to interfere with peptide stem amidation could therefore have potential for combatting *S. aureus*, including strains that are resistant to currently available antibiotics. Precise knowledge about the three-dimensional structure, organization and catalytic mechanism of the GatD/MurT complex is invaluable for fully exploiting this potential.

The crystal structure of GatD bound to MurT establishes an initial framework for probing the function of this enzyme complex and for developing strategies to intervene with amidation. The two proteins assemble into an elongated complex, with a large open space separating GatD from the middle domain of MurT. The previously identified catalytically active cysteine (GatD-C94)^19^ is located near the GatD/MurT interface, and is part of a catalytic triad that includes additional residues from both proteins (GatD-H189, MurT-D349). As our mutagenesis data confirm the importance of both GatD-C94 and MurT-D349 for catalysis while recent findings by Leisico and coworkers^24^ corroborate the crucial role of GatD-H189, the location of this putative catalytic triad nicely explains the requirement of the assembled complex for activity, and it is in agreement with the observed interface between the two proteins. Complex formation with an ATP analog revealed a nucleotide-binding site in MurT that is similar to those found in other Mur ligases. It is conceivable that the large open space separating the GatD/MurT catalytic triad and ATP binding site may accommodate a portion of the bulky Lipid II substrate. The SAXS experiments demonstrate that the GatD/MurT complex has a similar open conformation in solution, and they also show that small movements of the terminal regions towards each other are possible. Such movements might be more pronounced in the presence of Lipid II, as the enzyme might partially close around it during catalysis.

The hydrophobicity of Lipid II and the lack of suitable, commercially available less hydrophobic Lipid II alternatives did not allow us to effecitively pursue a complex of GatD/MurT bound to Lipid II. Soaking or co-crystallization experiments with soluble mimics of the peptide stem alone (e.g. glutamate, or commercially available peptides based on the *S. aureus* peptide stem sequence) did not result in complex formation, suggesting that additional portions of the Lipid II molecule are required for binding.

The *S. aureus* enzyme GatD/MurT likely belongs to a distinct group of enzymes that is strongly conserved throughout a subset of bacteria exhibiting a high degree of peptidoglycan amidation^16^, as suggested by conservation analysis^19^. The organisms that express GatD/MurT homologs include a number of established human pathogens, such as *Streptococcus pneumoniae*, for which the GatD/MurT enzyme complex was recently characterized^31^, and, likely, *Mycobacterium tuberculosis*. Knowledge about the structure and catalytic mechanism of GatD/MurT will therefore transcend the *S. aureus* field.

Structurally, GatD belongs to the family of glutamine amidotransferases (GATases), whose members are involved in a multitude of biosynthetical processes. While GATases exhibit a wide variety of substructure variants due to differing substrates or co-substrates, their core architecture is highly conserved, including that of GatD. The elongated C-terminal loop leading to an additional C-terminal α-helix (helix α7) is, however, unique to GatD. This segment contributes 36% of the dimer interface with MurT, and the length of the linker connecting it to the rest of the protein suggests that it may allow for a certain degree of flexibility. Thus, the linker could help mediate breathing of the complex, which would explain the observed differences in the overall GatD/MurT conformation between the crystal structure and the solution scattering data (Fig. 2).

While MurT lacks the N-terminal domain typically found in Mur ligases, the overall folds of the middle and C-terminal MurT domains agree very well with the equivalent domains in published structures of different Mur ligases from various organisms. Although the position of the observed ATP-binding pocket is strictly conserved throughout Mur ligases (Fig. 5), the angle between the middle and C-terminal domains varies significantly across different enzymes, indicating an inherent flexibility of this feature. The almost linear arrangement between the two MurT domains appears to be unique to MurT as all other known conformations of this two-domain segment are more closed in Mur ligases. The two most striking structural differences between MurT and related enzymes are the truncated N-terminal domain and the Cys_4_ Zinc-ribbon situated at the side of the middle domain. While the role of this last feature is unclear, it forms a very exposed structure protruding from the main bulk of the MurT middle domain. Zinc fingers of this class have been previously shown to provide a platform for protein-protein interactions^36^. The zinc finger is less conserved compared with many other features of GatD/MurT, and it appears to be present only in a subset of bacteria with putative *gatD/murT* genes.

The truncated N-terminus (with respect to other Mur ligases), on the other hand, is a well-conserved feature of MurT in different organisms. The closely related MurC-F enzymes catalyze the consecutive addition of amino acids of the peptide stem to UDP-activated MurNAc in the initial phase of peptidoglycan biosynthesis. The roughly 90 residues long N-terminal domain present in these enzymes plays an important role in substrate recognition by interacting with its UDP moiety. In contrast, MurT has a much shorter N-terminus of only 35 residues, which appears to be structurally flexible and poorly ordered as it could not be observed in the crystal structure. Furthermore, the positioning of the pyrophosphate linker in the UDP-conjugated substrates of ligand-bound structures of *S. aureus* MurE (PDB ID 4c12) and *Pseudomonas aeruginosa* MurF (PDB ID 4cvk) coincides with the boundary between their N-terminal and middle domains. In the GatD/MurT substrate Lipid II, the pyrophosphate linkage connects the sugar moiety to the undecaprenyl membrane anchor, possibly marking the position at which Lipid II emerges from the membrane. Hence, a UDP-MurNAc-binding N-terminal domain is not only unnecessary, it would also likely clash with the plasma membrane.

How does Lipid II bind to GatD/MurT, and how does catalysis proceed? Prompted by the drastically altered solubility of the protein observed by SAXS upon minimal substrate mimic addition (Supplementary Fig. S2) and based on previously published data revealing that MurT is able to replace MurE *in vitro*^19^ as well as on alignments with the substrate-bound structures of MurE and MurF, which consistently show closed conformations, an analogous structural rearrangement upon substrate binding can be modeled for MurT. To generate this model, the C-terminal domains of MurE or MurF were superimposed with that of MurT, and the MurT middle domain was then moved to fit those of MurE or MurF, producing a more closed conformation of MurT that mimics those present in the other two enzymes (Fig. 7). If the GatD structure is then added to the modified MurT using the known GatD/MurT interface, it becomes clear that the distance between the MurT middle domain and GatD is drastically reduced. The gap between GatD-C94 and the γ-phosphate of AMPPNP diminishes from over 40 Å to just over 20 Å. In such a putative more closed conformation of GatD/MurT, the peptide stem of Lipid II would be sandwiched between the MurT middle and C-terminal domains in linear continuation of the triphosphate axis of the nucleotide (Fig. 7b). It is conceivable that channeling of nascent ammonia from the active site of GatD to the muropeptide substrate in MurT, which would then lie in close proximity to the nucleotide, may be enabled by such a closed conformation. The DNADD motif, which was previously suggested to be involved in the engagement of L-lysine in the muropeptide sequence^19^, indeed comes into close proximity with the modeled MurE product, which was also extracted from PDB ID 4c12. Due to the direct backbone connection with MurT-D349, the relative position of the residues forming the putative catalytic triad may be influenced by Lipid II binding to the enzyme. Thus, hydrolysis of glutamine in the absence of an acceptor substrate and hence escape and accumulation of ammonia in the cell could be avoided.

The model presented here was obtained by domain superimposition with known substrate-bound structures of homologous proteins, and it is only meant to approximate the possible conformations of ligand-bound GatD/MurT and the Lipid II substrate. While the superimposition predicts a closed conformation of substrate-bound GatD/MurT, some residues located at the newly formed interface between the MurT C-terminal and middle domains clash both with the respective other domain and with the modeled MurE ligand, and this demonstrates that additional movements in the protein are required to properly accomodate the substrate. As the physiologic substrate of MurT, Lipid II, differs from that of MurE, it would not be surprising if the actual active conformation of GatD/MurT may be a few degrees more open to allow accommodation of the large Lipid II molecule. The precise conformation of a Lipid II-bound complex of GatD/MurT is still unknown and likely somewhat different from the one shown in Fig. 7. We therefore believe that performing in-depth molecular docking calculations would be inappropriate at this time.

Our model suggests that the mechanism of Lipid II amidation likely starts with an ATP-bound, open conformation of GatD/MurT similar to that observed in our crystal structure. A domain rearrangement between the two MurT domains to generate a more closed conformation could be triggered by the binding of the substrate peptide, and this would enable both the hydrolysis of glutamine by GatD through movement of the triad residues and the correct positioning of the free carboxylate of D-iso-glutamate in the peptide stem of Lipid II with respect to ATP, thus allowing for a concerted amidation reaction. Of course, this hypothesis needs to be tested in future experiments.

Peptidoglycan is characteristically modified in many Gram-positive pathogens and amidation is known to affect the level of methicillin resistance^21,37^ and to contribute to vancomycin susceptibility in *S. aureus*^38^, and thus inhibition of the GatD/MurT complex may represent a strategy to combat *S. aureus* and perhaps also other related bacterial pathogens that amidate their peptide stem in a similar manner. The nucleotide-binding region of MurT may provide a good base for developing multiple-target inhibitors, owing to the similarity of this site with those of other Mur ligases. However, as most monomeric Mur ligases are also present in beneficial bacteria such as *E. coli* or other components of our gut microbiota, it may be desirable to develop drugs more specifically aimed at GatD/MurT. As the GatD/MurT interface is highly conserved and likely unique to complexes between these two proteins, interfering with the interaction between GatD and MurT could selectively inactivate the amidation reaction. It is conceivable that small molecules that mimic components of the interface could serve to block the reaction carried out by the GatD/MurT complex.

## Materials and Methods

### Expression and purification

A pET21b vector (Novagen) containing the *gatD/murT* operon for co-expression of untagged MurT with GatD as a C-terminal His-tag fusion protein [1] was introduced into E. coli BL21 (Promega) cells. The bacteria were grown in LB-medium (75 µg/mL ampicillin) at 37 °C. At an OD_600_ of 0.6, the cultures were cooled to 30 °C and 0.25 mM IPTG was added for induction. Cells were harvested by centrifugation after 5 hours and stored at −20 °C until purification.

Frozen pellets were resuspended in lysis buffer (50 mM Tris pH 8.5 at 4 °C, 300 mM NaCl, 10 mM imidazole, 1 mM DTT). Next, 200 µg/mL lysozyme, 1:10000 DNAse I (ThermoFisher) and 1 mg RNAse A (Sigma) were added and cells were incubated on ice for 30 min. Following lysis by sonication, the lysate was clarified by centrifugation and added to a Ni-NTA-agarose slurry (Novagen). The mixture was incubated on a tilt shaker at low rpm for 18 h at 4 °C and subsequently transferred to a EconoPac gravity flow column (BioRad). Following a wash with lysis buffer, weakly bound material was removed by washing with lysis buffer containing 50 mM imidazole, and the target protein was then eluted with lysis buffer containing 350 mM imidazole. Buffer exchange to SEC I buffer (50 mM Tris pH 8.2 @ 4 °C, 500 mM NaCl, 50 mM MgCl_2_, 5 mM KCl, 5 mM DTT) was carried out directly afterwards using PD-10 desalting columns (GE). Sample homogeneity was achieved by means of two subsequent iterations of preparative size exclusion chromatography using a HiLoad 26/60 column packed with Superdex 200 column material (GE Healthcare). In the second iteration, 5 mM TCEP was substituted for DTT as a reducing agent (Buffer SEC II). Throughout the purification, sample purity was assessed by SDS-PAGE. The protein was freshly prepared for each analysis.

### Crystallization and structure determination

Crystals of GatD/MurT were grown in a sitting-drop vapor diffusion setup using protein concentrations of 2–5 mg/mL in a broad range of Tris-buffered conditions containing PEG8000, MgCl_2_ and glycerol. The conditions from which the crystals for this study were obtained are 0.1 M Tris pH 8.6, 40% (w/v) PEG8000, 0.35 M MgCl_2_ for the native data, 0.1 M Tris pH 9.0, 26.7% (w/v) PEG8000, 0.35 M MgCl_2_ for the thiomersal derivative, and 0.1 M Tris pH 9.1, 18% (w/v) PEG8000, 14% glycerol, 0.35 M MgCl_2_ for the AMPPNP soak, respectively. Cryoprotection was performed by adding 20% MPD or 25% glycerol to the reservoir solution, and crystals were flash-cooled in liquid nitrogen. X-ray data were collected at beamlines X10SA and X06DA of the Swiss Light Source (SLS) in Villigen (Switzerland). Data were integrated and reduced using the XDS program package^39^.

### Crystal derivatization

Crystals were derivatized with 10 mM thiomersal for phase determination and a mixture of 2.1 mM of the ATP analogue β,γ-imidoadenosine 5′-triphosphate (AMPPNP), 2.1 mM of the glutamine analogue 6-Diazo-5-oxo-L-norleucine (DON), and 2.5 mM UDP-MurNAc-L-Ala-D-Glu-γ-L-Lys-D-Ala-D-Ala for evaluating complex formation. Of the latter three compounds, only AMPPNP was found to bind to the crystals based on the inspection of difference electron density maps. Derivatization was performed by transferring native crystals into drops of otherwise equal composition containing the compound to be soaked. After soaking times of 10 to 30 min, crystals were cryoprotected and flash-cooled in liquid nitrogen.

### Phasing and initial model building

A dataset obtained from a crystal derivatized with 10 mM thiomersal was used to extract initial phase information using the single isomorphous replacement with anomalous scattering (SIRAS) approach. The derivative data were scaled against the native data using CAD and Scaleit of the CCP4 program suite^40^. Using the program suite autoSHARP^41,42^, a substructure of 13 mercury sites was determined, followed by heavy atom refinement and density modification. Initial automated model building was performed using the AutoBuild Wizard^43^ in the PHENIX program suite prior to transfer to the native data set.

### Model building and refinement

Manual model building was carried out in Coot^44^ and alternated with reciprocal space refinement using the programs REFMAC5^45^ and PHENIX.REFINE^46^. The data set collected from crystals soaked with AMPPNP was phased by molecular replacement using the program PHASER^47^. Prior to model building, simulated annealing was performed using PHENIX.REFINE in order to avoid model bias from the original dataset.

### SAXS

Small-angle X-ray scattering (SAXS) data were collected at beamline P12 at the German Electron Synchrotron (DESY) in Hamburg, Germany. Triplicates of a two-fold dilution series of GatD/MurT in SEC II buffer ranging from 0.5 to 8 mg/mL were recorded and extrapolated to zero concentration to remove possible concentration effects using the PRIMUS interface of the ATSAS program suite^48^. Rigid-body refinement of the native crystal structure was carried out with the program SREFLEX^49^. Synthetic scattering profiles for comparison with the experimental data were generated using CRYSOL^50^. SAXS experiments were repeated in the presence of increasing concentrations of the ATP analog AMPPNP (10 µM–5 mM) and the soluble Lipid II mimic UDP-MurNAc-L-Ala-D-Glu-γ-L-Lys-D-Ala-D-Ala (8 µM–1 mM).

### Mutagenesis and *in vitro* amidation assay

Site-directed mutagenesis was performed according to the manufacturer´s instructions using plasmid pET21-*murT/gatD* as the template to generate active site mutants GatD-C94S and MurT-D349N (QuikChange Lightning Site-Directed Mutagenesis Kit, Agilent) and to generate mutations in the ATP binding site (MurT mutants T60A, E108A, N267Y and double mutant T60A E108A; Q5 site-directed mutagenesis kit, New England Biolabs). Proteins were expressed and purified and impact of mutations on enzyme activity were tested as previously described^19^.

### Circular dichroism (CD) spectroscopy

CD spectroscopy experiments were performed on a JASCO J-720 spectrometer under nitrogen flow. Protein concentration was set to 0.3 mg/mL in ten-fold diluted SEC II buffer. The resulting spectra were corrected for concentration, protein size and cuvette thickness. Mutant protein spectra were compared with the wildtype.

### Thermal shift assay (TSA)

TSA was performed on a QuantStudio 5 real-time PCR cycler (Applied Biosystems, Thermo Fisher Scientific). Wildtype and mutant GatD/MurT were supplied at 0.3 mg/mL and Protein Thermal Shift Dye^TM^ (Applied Biosystems, Thermo Fisher Scientific) was added as a TSA fluorophore. The samples were equilibrated at 4 °C and gradually heated to 95 °C over 30 min while monitoring dye fluorescence. The averaged protein melting temperature for the wildtype and each mutant was derived from the melting curve inflection point using seven technical replicates and the Protein Thermal Shift^TM^ Software v1.3 (Applied Biosystems, Thermo Fisher Scientific).

### Homology searches, conservation analysis and interface mapping

Sequence-based conservation analysis was performed using ConSurf^34^. Herein, an initial search was automatically performed separately for GatD and MurT using BLAST (https://blast.ncbi.nlm.nih.gov/) and a multiple sequence alignment (MSA) of 145 sequences each was generated using MAFFT^51^. The resulting conservation scores were then projected onto the GatD/MurT crystal structure. Color coding of conservation was done by setting the average conservation score of the alignment to zero. More or less conserved residues were then colored according to a color ramping scheme, with dark blue and red indicating highest and lowest conservation scores, respectively. Structural homology searches were conducted at a secondary structure level using the HHPRED tool implemented in the bioinformatics toolkit of the Max-Planck-Institute for Developmental Biology in Tübingen^52^.

Additionally, previously postulated putative^19^ and confirmed^31^ homologous proteins were aligned separately, using the ClustalΩ algorithm^53^.

Finally, in order to identify related proteins based on their entire structure and quantify the structural similarities, a DALI search^25^ was performed with the final crystal structure against the entire Protein Data Bank (PDB) repository at www.rcsb.org.

In order to classify contact surfaces observed in the crystal, the crystallographic assembly was subjected to analysis by the PISA^28^ and EPPIC^29^ servers.

### Data deposition

The GatD/MurT structures described here have been deposited with the Protein Data Bank (www.rcsb.org) with PDB IDs 6GS2 (unliganded GatD/MurT) and 6H5E (complex with AMPPNP).

## Electronic supplementary material

Supplementary Information
