# Author Correction: Structural basis of cell wall peptidoglycan amidation by the GatD/MurT complex of *Staphylococcus aureus*

**DOI:** 10.1038/s41598-018-37082-9

**Published:** 2018-12-21

**Authors:** Erik R. Nöldeke, Lena M. Muckenfuss, Volker Niemann, Anna Müller, Elena Störk, Georg Zocher, Tanja Schneider, Thilo Stehle

**Affiliations:** 1 Interfaculty Institute of Biochemistry, University of Tübingen, D-72076 Tübingen, Germany; 2 Institute for Pharmaceutical Microbiology, University of Bonn, D-53115 Bonn, Germany; 3 Vanderbilt University School of Medicine, Nashville, Tennessee 37232 USA; 4 Present Address: Department of Biochemistry, University of Zurich, CH-8057 Zurich, Switzerland; 5 Present Address: Hain Lifescience GmbH, D-72147 Nehren, Germany

Correction to: *Scientific Reports* 10.1038/s41598-018-31098-x, published online 28 August 2018

In Fig. 1A, a carbonyl group is missing in the schematic drawing of the reaction product. The correct Fig. 1 appears below.

**Figure 1:**
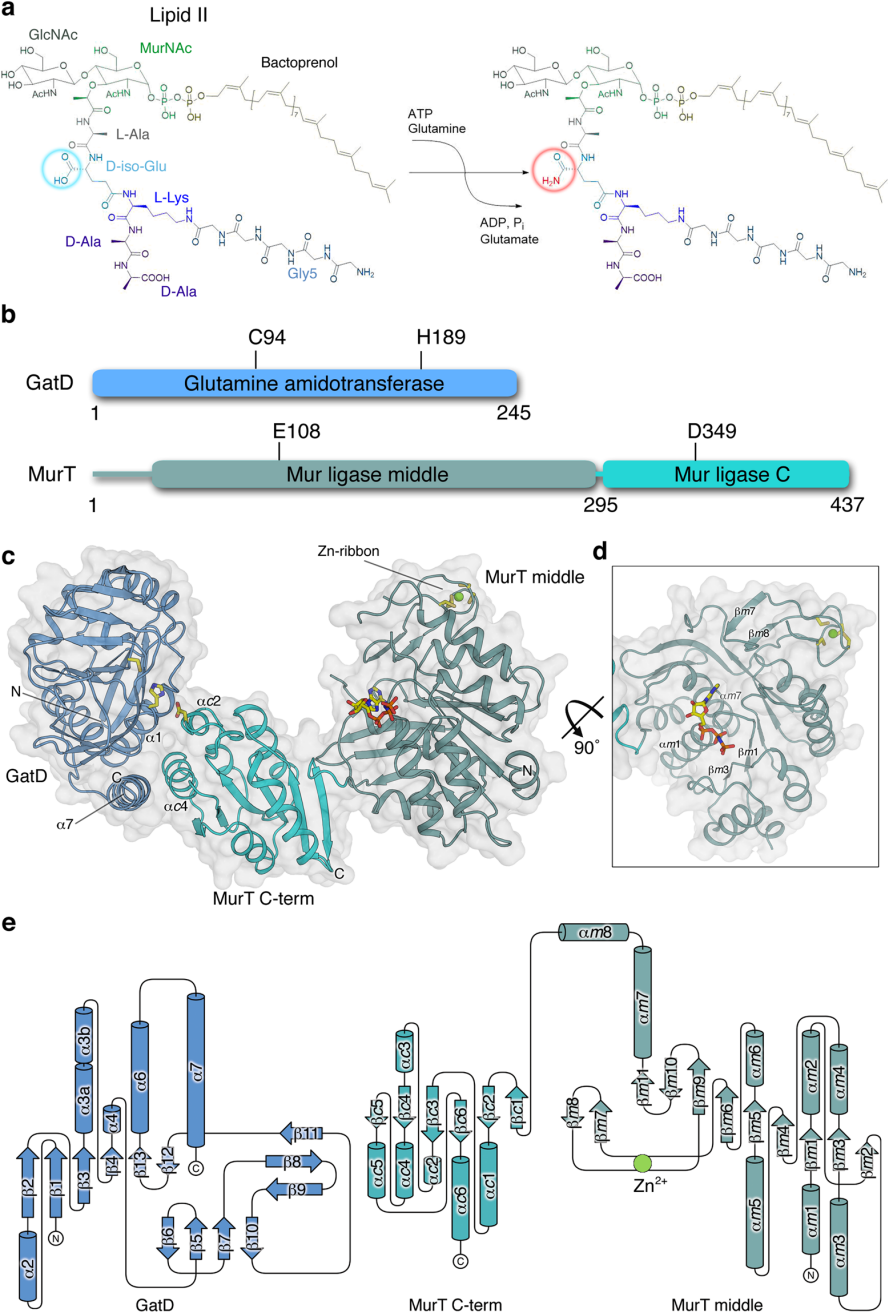
Overall structure and organization of the GatD/MurT complex. (**a**) Reaction catalyzed by GatD/MurT. The free α-carboxyl of D-iso-glutamate in the peptide stem is amidated in a glutamine- and ATPdependent reaction. (**b**) Schematic overview of GatD and MurT proteins. GatD consists of a single glutamine amidotransferase (GATase) domain with a cysteine at position 94 as the active residue and a histidine at position 189 as a component of the catalytic triad^19^. MurT is composed of two domains: a Mur ligase middle domain (MurT middle) containing the canonical ATP binding site and, surprisingly, a ribbon-type Zinc finger, and a C-terminal Mur ligase domain (MurT C-term). MurT residue glutamate 108 participates in ATP hydrolysis, and aspartate 349 forms the third residue in the putative catalytic triad. (**c**) Overview of the GatD/MurT structure. GatD and MurT form a boomerang-shaped complex, with GatD contacting the MurT C-term domain through contacts that are in part mediated by helix α7 of GatD. Catalytic triad residues GatD-C94, GatD-H189, MurT-D349 and the bound nucleotide AMPPNP are shown in stick representation. The zinc ion in the Cys_4_ zinc ribbon of MurT is shown as a green sphere, and the four cysteine residues ligating it are shown as sticks. (**d**) Tilted view of the MurT middle domain to show the central β-sheet and the bound AMPPNP and its surrounding secondary structure elements, as well as the zinc ribbon. (**e**) Topological representation of the GatD/MurT architecture. Secondary structure nomenclature of GatD was done according to Leisico *et al*.^24^. As the short helices α1 and α5 in the isolated GatD structure do not conform to helical geometry in our complex, they were not assigned. The MurT domains were assigned separately with the prefixes *m* and *c* indicating the middle and C-terminal domains, respectively. The drawing was generated with TopDraw^54^.

