# Supplementary material for: Structural basis of cell wall peptidoglycan amidation by the GatD/MurT complex of Staphylococcus aureus
Source: Sci Rep. 2018 Aug 28;8:12953. doi: 10.1038/s41598-018-31098-x (PMC6113224; doi:10.1038/s41598-018-31098-x)
Supplement: Supplementary file 1 — Supplementary Information [file 41598_2018_31098_MOESM1_ESM.pdf]

Supplementary Information for:

Structural basis of cell wall peptidoglycan amidation by the GatD/MurT complex of *Staphylococcus aureus*

Erik R. Nöldeke[1], Lena M. Muckenfuss[1,4], Volker Niemann[1,5], Anna Müller[2], Elena Störk[1], Georg Zocher[1], Tanja Schneider[2], Thilo Stehle[1,3]

[1]Interfaculty Institute of Biochemistry, University of Tübingen, D-72076 Tübingen, Germany

[2]Institute for Pharmaceutical Microbiology, University of Bonn, D-53115 Bonn, Germany

[3]Vanderbilt University School of Medicine, Nashville, Tennessee 37232, USA

[4]Current address: Department of Biochemistry, University of Zurich, CH-8057 Zurich, Switzerland

[5]Current address: Hain Lifescience GmbH, D-72147 Nehren, Germany

Table S1. Melting temperatures (T_m) for GatD/MurT wild type and mutants

Thermal Shift Assay-derived melting temperatures for GatD/MurT wild type and mutants (triad: GatD-C94S, MurT-D349N; ATP-binding residues T60A, E108A, N267Y in MurT). Melting temperatures were obtained from the melting curve (Supplementary Figure S6) inflection points in seven replicates. The standard error of the mean is shown.

Mutant	T_m
wt	45.43 ˚C ± 0.03 ˚C
GatD-C94S	43.42 ˚C ± 0.04 ˚C
MurT-D349N	42.14 ˚C ± 0.05 ˚C
MurT-T60A	45.80 ˚C ± 0.09 ˚C
MurT-E108A	45.98 ˚C ± 0.05 ˚C
MurT-N267Y	46.17 ˚C ± 0.07 ˚C

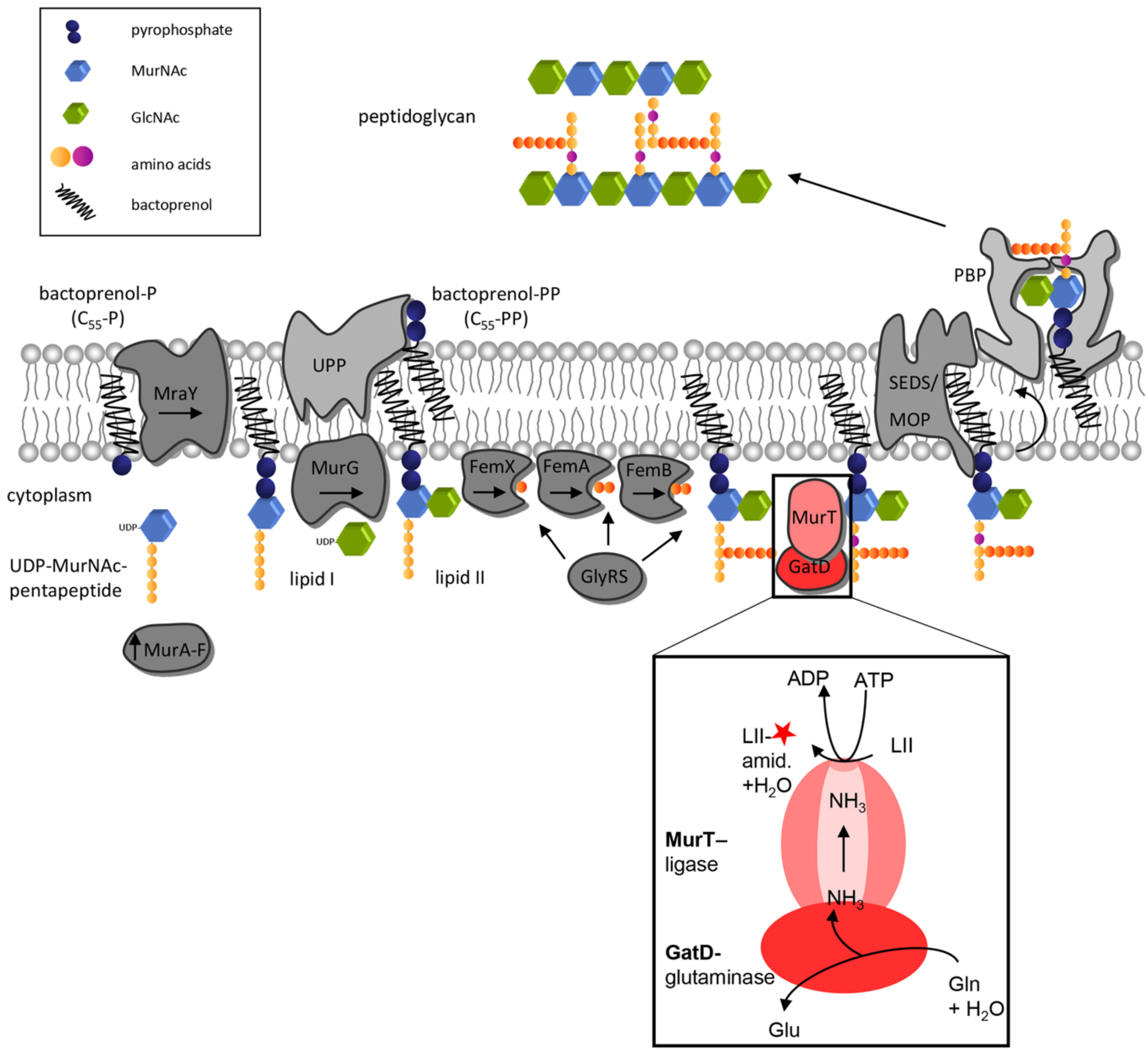

Figure S1. Schematic depiction of the GatD/MurT-catalyzed amidation during peptidoglycan biosynthesis in S. aureus

The GatD/MurT bi-enzyme complex uses glutamine as the primary nitrogen donor, and ammonia is shuttled from the GatD glutaminase active site to the active site of the MurT ligase. MurT finally catalyzes the amidation in an ATP-dependent fashion to the acceptor substrate Lipid II. Figure modified from Münch et al. (2012). PLoS Pathog. 8:e1002509.

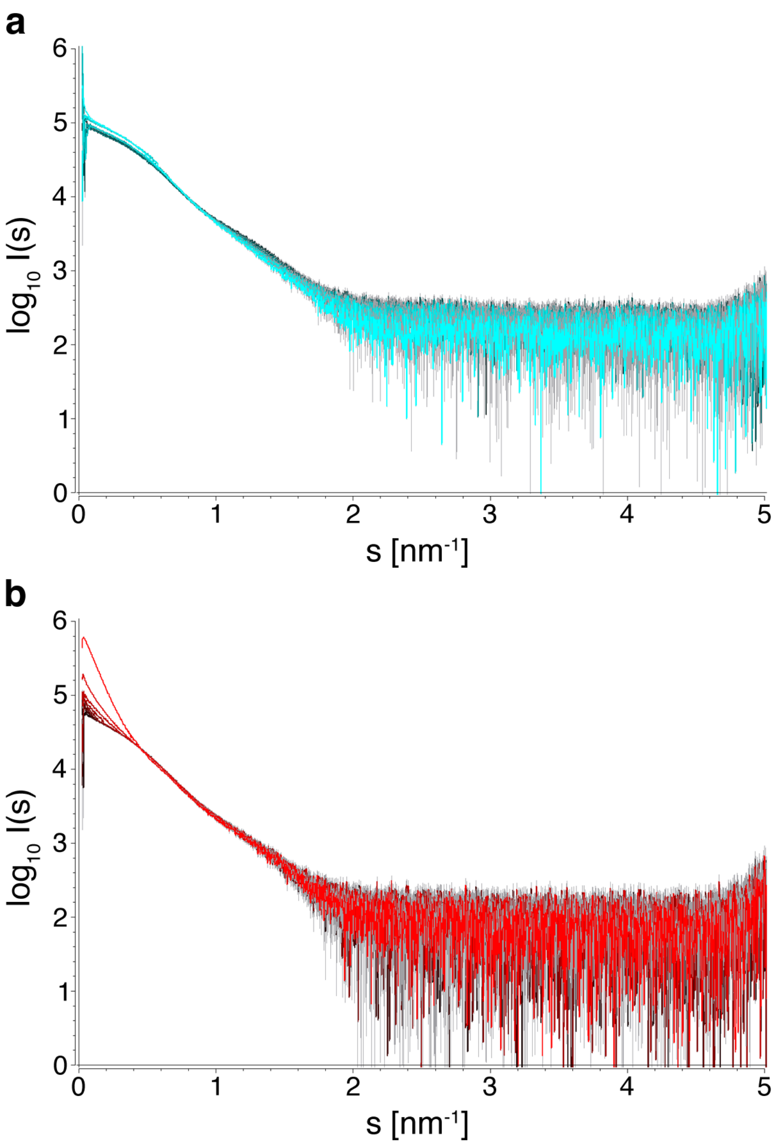

Figure S2. Ligand-induced aggregation of GatD/MurT

Both the addition of AMPPNP (a) and the soluble Lipid II analog UDP-MurNAc-L-Ala-D-Glu-γ-L-Lys-D-Ala-D-Ala (b) lead to concentration-dependent aggregation of GatD/MurT. (a) Protein-concentration-corrected SAXS scattering profiles of GatD/MurT in absence (black) and presence (cyan gradient) of increasing concentrations of AMPPNP up to 5 mM in a twofold dilution series. The increasing signal at very low scattering angles as well as the loss of signal at intermediate scattering angles of around 1.5 nm-1 are clear indicators of moderate aggregation. (b) Titration of UDP-MurNAc-pentapeptide from none (black) to 1 mM (red) leads to strong aggregation, visible by the extreme increase of signal intensity at low scattering angles. While these data do not allow ab-initio or rigid body modelling, they clearly show a strong concentration-dependent effect of both substrate analogues on the solubility of GatD/MurT. One explanation for the reduced solubility would be a ligand-triggered domain rearrangement in the enzyme complex.

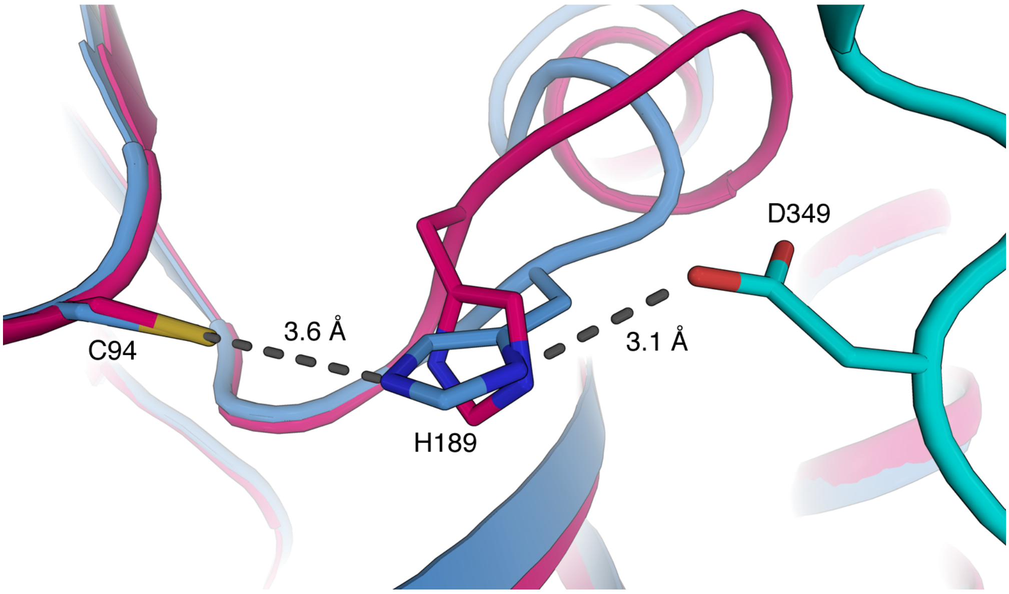

Figure S3. Orientation of GatD-H189 is dependent on GatD/MurT interaction.

Overlay of the GatD/MurT catalytic triad with the recently published structure of GatD without its interaction partner MurT (PDB ID 5n9m, shown in pink). The loop containing GatD-H189 adopts a different conformation in the absence of MurT, shifting the H189 sidechain out of the linear assembly required for efficient proton relay. The interactions provided by the complex formation with MurT are thus required in order to form an active conformation.

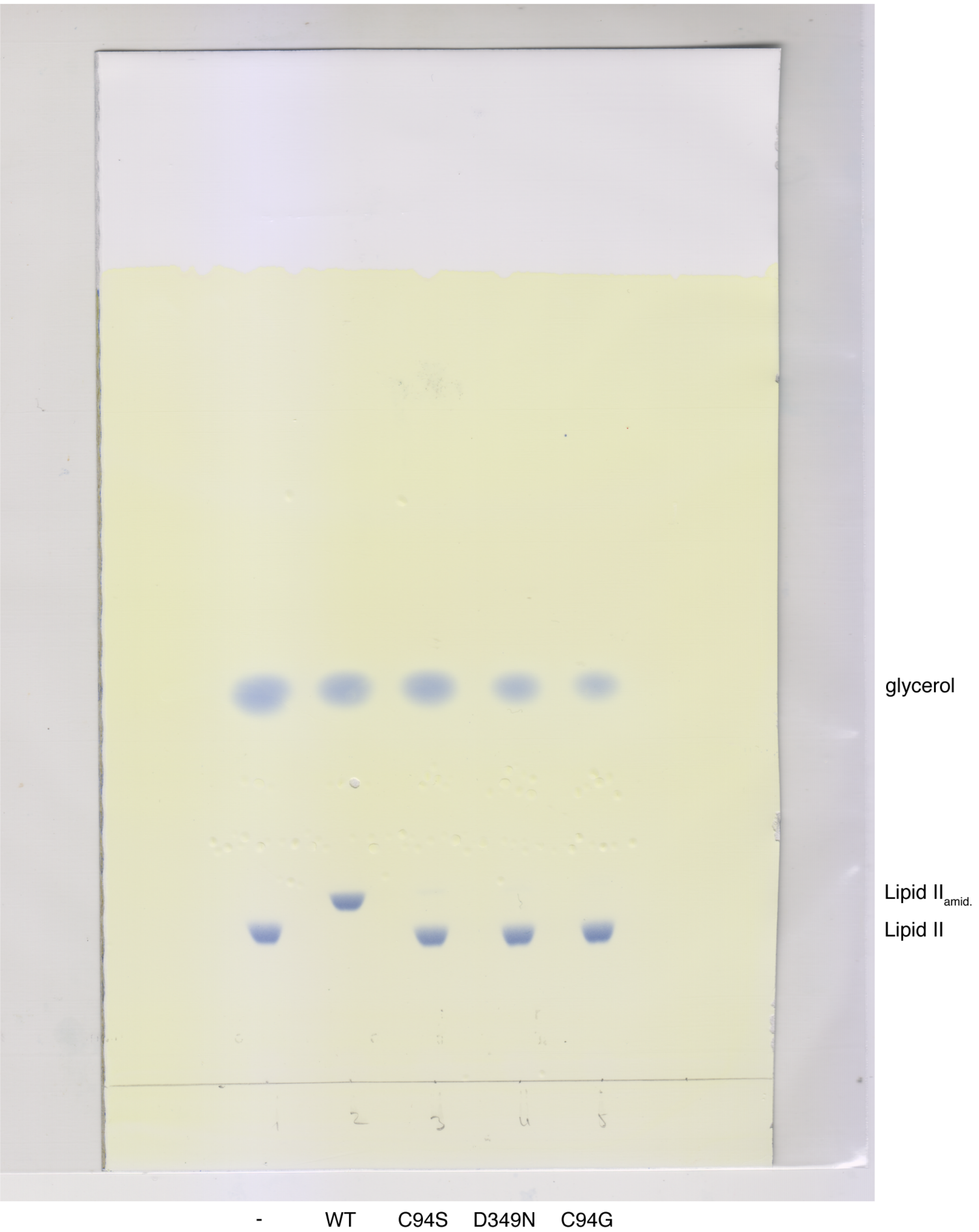

Figure S4. Uncropped representation of TLC from Fig. 4c

Uncropped version of thin-layer chromatography of Lipid II amidation assays performed with wild type GatD/MurT and putative catalytic triad mutants. Used in Fig. 4c.

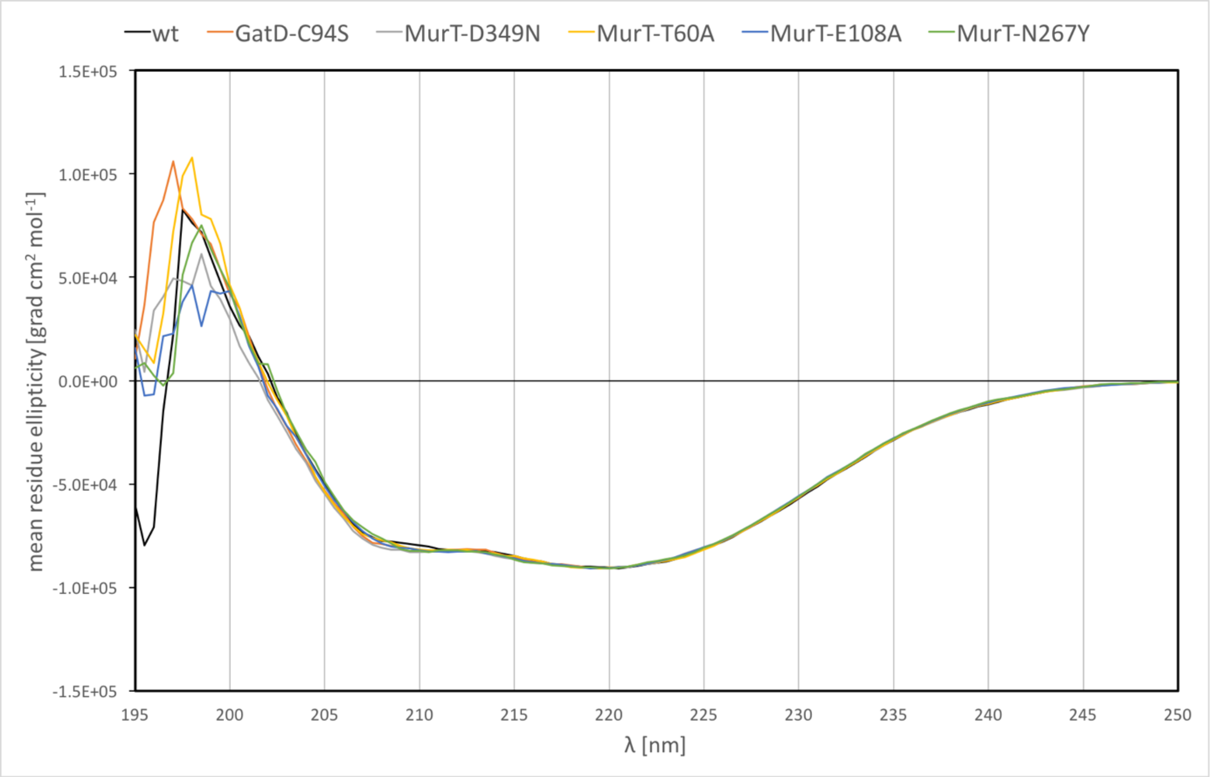

Figure S5. Circular Dichroism (CD) spectra for GatD/MurT wild type and mutants

Normalized CD spectra of GatD/MurT wild type and mutants (triad: GatD C94S, MurT D349N; ATP-binding: MurT T60A, E108A, N267Y). Spectral comparison suggests indistinguishable folds. The different proteins are colored according to the scheme at the top of the figure.

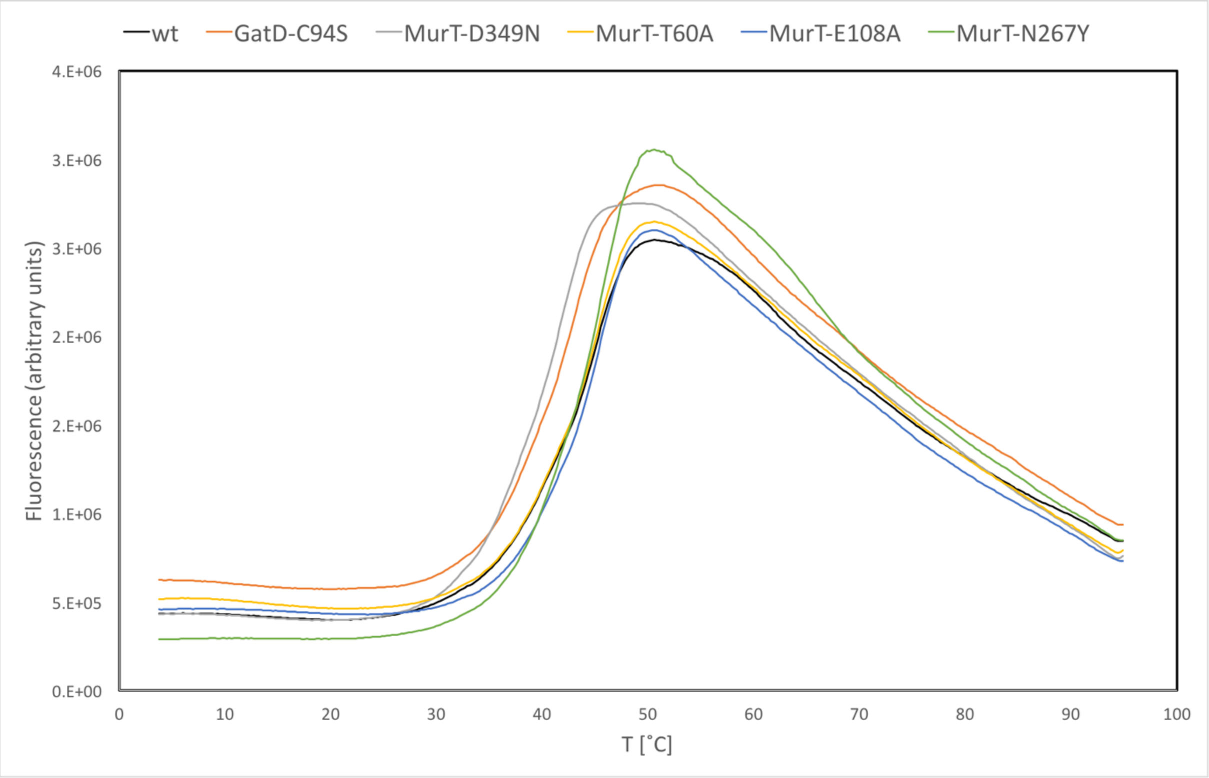

Figure S6. Thermal Shift Assay melting curves for GatD/MurT wild type and mutants

Representative melting curves for GatD/MurT wild type and mutants (triad: GatD-C94S, MurT-D349N; ATP-binding residues T60A, E108A, N267Y in MurT). Color coding as in Supplementary Figure S5, the inflection point derived melting temperatures (T_m) are shown in Supplementary Table S1. The different proteins are colored according to the scheme at the top of the figure.

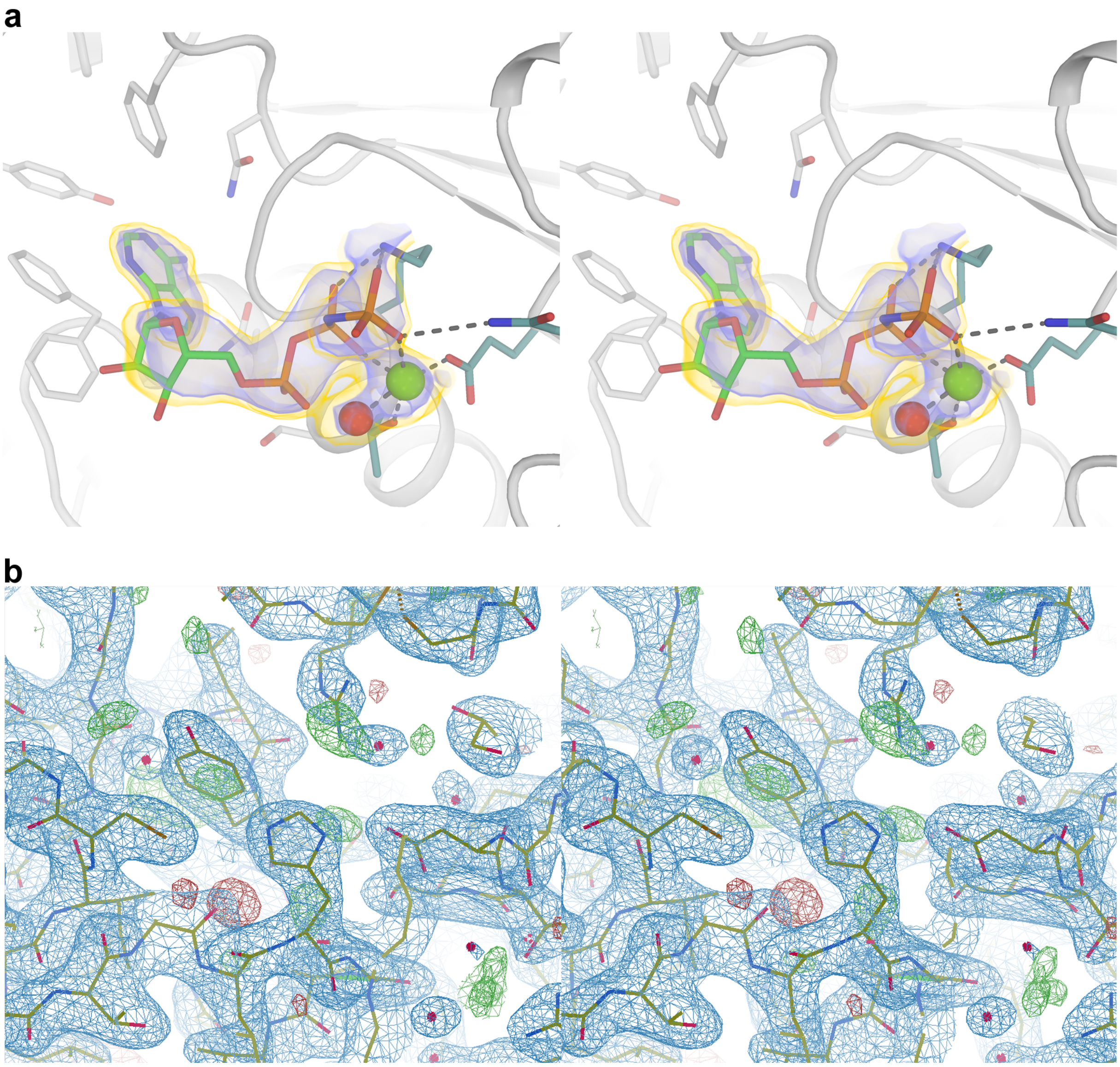

Figure S7. Representative sections of electron density

Wall-eyed stereo views of electron density from GatD/MurT. (a) Unbiased simulated annealing omit mF_o-DF_c difference density showing the bound AMPPNP and complexed Mg^{2+} ion contoured at 3 σ (blue) and at 2.5 σ (yellow). The view is the same as in Fig. 5a. (b) Representative density at the end of refinement. $2mF_o$-DF_c map of the GatD/MurT catalytic triad is shown in blue and contoured at 1.5 σ, the mF_o-DF_c difference density contoured at 3 σ is shown green for positive and red for negative values.

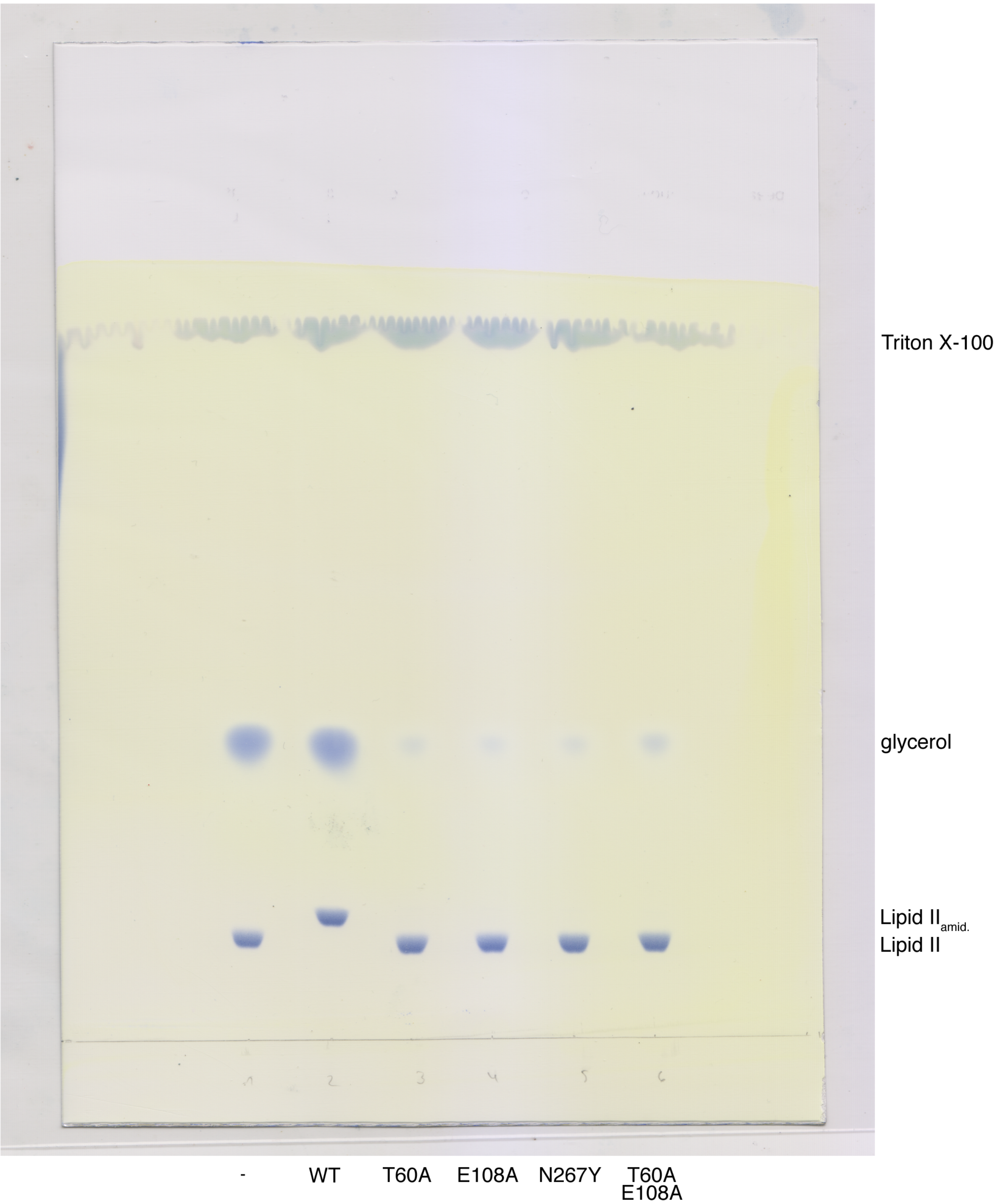

Figure S8. Uncropped representation of TLC from Fig. 5c

Uncropped version of thin-layer chromatography of Lipid II amidation assays performed with wild type GatD/MurT and putative ATP-binding site mutants. Used in Fig. 5c.
